# Chronic dysfunction of Stromal interaction molecule by pulsed RNAi induction in fat tissue impairs organismal energy homeostasis in *Drosophila*

**DOI:** 10.1038/s41598-019-43327-y

**Published:** 2019-05-06

**Authors:** Yanjun Xu, Annika F. Borcherding, Christoph Heier, Gu Tian, Thomas Roeder, Ronald P. Kühnlein

**Affiliations:** 1 Max-Planck-Institut für biophysikalische Chemie, Research Group Molecular Physiology, Am Faβberg 11, D-37077 Göttingen, Germany; 2 Max-Planck-Institut für biophysikalische Chemie, Department of Molecular Developmental Biology, Am Faβberg 11, D-37077 Göttingen, Germany; 3 Institute for Diabetes and Obesity, Helmholtz Diabetes Center, Helmholtz Zentrum München, D-85764 Neuherberg München, Germany; 4 University of Graz, Institute of Molecular Biosciences, Humboldtstrasse 50/2.OG, A-8010 Graz, Austria; 5 BioTechMed Graz, Graz, Austria; 6 Christian-Albrechts University Kiel, Zoology, Molecular Physiology, 24098 Kiel, Germany; 7 Airway Research Center North (ARCN), Member of the German Center for Lung Research (DZL), Kiel, Germany

**Keywords:** RNAi, Homeostasis, Metabolic disorders

## Abstract

Obesity is a progressive, chronic disease, which can be caused by long-term miscommunication between organs. It remains challenging to understand how chronic dysfunction in a particular tissue remotely impairs other organs to eventually imbalance organismal energy homeostasis. Here we introduce RNAi Pulse Induction (RiPI) mediated by short hairpin RNA (shRiPI) or double-stranded RNA (dsRiPI) to generate chronic, organ-specific gene knockdown in the adult *Drosophila* fat tissue. We show that organ-restricted RiPI targeting *Stromal interaction molecule (Stim)*, an essential factor of store-operated calcium entry (SOCE), results in progressive fat accumulation in fly adipose tissue. Chronic SOCE-dependent adipose tissue dysfunction manifests in considerable changes of the fat cell transcriptome profile, and in resistance to the glucagon-like Adipokinetic hormone (Akh) signaling. Remotely, the adipose tissue dysfunction promotes hyperphagia likely via increased secretion of Akh from the neuroendocrine system. Collectively, our study presents a novel *in vivo* paradigm in the fly, which is widely applicable to model and functionally analyze inter-organ communication processes in chronic diseases.

## Introduction

Energy homeostasis is pivotal to life. It is well-established that long-term positive energy balance drives obesity (reviewed in^1,2^), which is defined as abnormal fat accumulation causative for a number of human diseases^3^.

The prevalence of obesity in children and adults has substantially increased around the world since 1980^4^. Moreover, over the past decades, no country has been successful in combating the obesity pandemic^4^. It is still under debate whether the increased supply of high calorie food^5^ combined with sedentary life style^6,7^ alone could explain the spread of obesity. But it is clear that the primary driver of obesity is the mismatch of energy intake and expenditure. Specifically, the maintenance of energy balance requires coordination of the major energy handling tissues such as liver (reviewed in^8^) and adipose tissue (reviewed in^9^) in mammals. Next to storing energy, adipose tissue also senses the energy status and communicates via adipokines to affect energy intake^10^ and expenditure^11^. Therefore, it is vital to understand how energy storage tissue regulates organismal energy balance both in an organ-specific manner and systemically.

Much like mammals, flies also have an energy storage tissue called fat body, which functions similar to liver and white adipose tissue (reviewed in^12^). Importantly, the majority of extra energy in mammals and flies is deposited as triacylglycerols (TAGs) in adipose tissue intracellular organelles called Lipid Droplets (LDs) (reviewed in^9,12,13^). Notably, a number of lipid metabolism effectors and regulators has been found to be conserved from fly to man^14,15^. One of these regulatory pathways is the store-operated calcium entry (SOCE), a major determinant of the versatile second messenger intracellular Ca^2+^ (iCa^2+^). One core component of SOCE is the endoplasmic reticulum (ER) calcium sensor Stromal interaction molecule (abbreviated Stim in fly, STIM1 in mammals)^15,16^.

In non-excitable cells, the canonical G protein-coupled receptor (GPCR) signaling generates inositol 1,4,5- trisphosphate (IP3) in the cytosol^17^, which stimulates a first wave of Ca^2+^ release from ER stores to the cytoplasm by activating the calcium channel-receptor IP3R (reviewed in^18^). Stim/STIM1 senses the ER calcium depletion and re-localizes to ER-plasma membrane junctions, where it interacts with plasma membrane calcium channel proteins (called olf186-F in flies and Calcium release-activated calcium channel protein 1 (ORAI1) in mammals) to form active Ca^2+^ release-activated Ca^2+^ channels (CRACs), thereby ultimately facilitating the extracellular calcium entry^16,19–23^. With this iCa^2+^ increase, SOCE regulates a plethora of cellular processes over a wide temporal spectrum (reviewed in^24^). We previously used acute knockdown of *Stim* in the fat body to identify SOCE as an adiposity regulator in flies^15,25^. A recent study confirmed that also mammalian STIM1 regulates lipid metabolism in liver and muscle^26^. Moreover, the plasma membrane translocation of STIM1 required for normal SOCE is impaired in hepatocytes of obese mice, which causes further metabolic dysfunction^27^. While fat accumulation in response to acute SOCE interference is well established, the adverse consequences of chronic iCa^2+^ malfunction are unknown as Stim/STIM loss-of-function is lethal in mice^26^ and in flies^28^, while extended tissue-specific *Stim* knockdown using a temperature-controlled expression system suffers from technical limitations^15^.

Therefore, we developed a novel strategy called short-hairpin or double-stranded RNAi Pulse Induction (RiPI) to achieve tissue-specific, chronic knockdown of genes of interest exemplified by *Stim* in adult fly fat body. We demonstrate that the siRNAs generated by a short-term RiPI are long-lasting, which chronically reduces the expression of *Stim* mRNA in adult fly fat tissue and consequently leads to progressive severe obesity in flies. We further provide evidence that chronic knockdown of *Stim* not only causes the tissue-autonomous dysfunction of lipid mobilization in response to Adipokinetic hormone (Akh) and reduced insulin signaling in fly fat body, but also remotely controls hyperphagia. Our results suggest that chronic dysfunction of Stim/SOCE in the energy storage tissue can dramatically modulate the systemic regulatory network, which drives the organismal energy imbalance.

## Results

### Chronic *in vivo* gene knockdown by RNAi Pulse Induction (RiPI) in adult fly storage tissue

**Figure 1:**
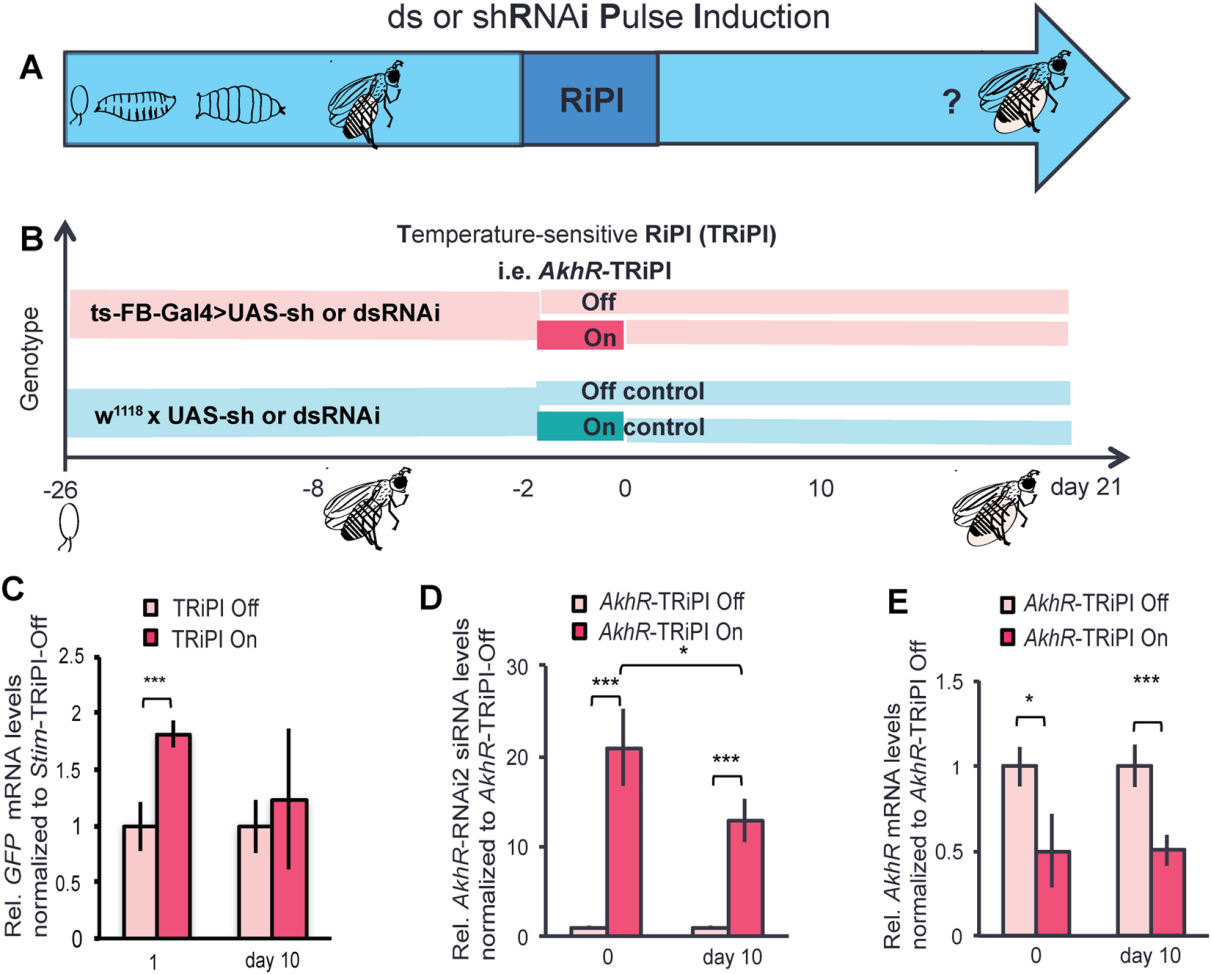
Transient RNAi Pulse Induction causes chronic gene knockdown in adult fly adipose tissue. (**A**) Overview of double-strand (ds) or short-hairpin (sh) **R**NA**i P**ulse **I**nduction (**RiPI**) regimen for chronic gene impairment in adult flies. (**B**) Detailed scheme showing the time period (from 18 °C to 29 *°*C for 38–48hs, starting at day 6 after eclosion of adult flies) of Temperature-sensitive **RiPI** (**TRiPI**, based on TARGET system) on target genes, i.e. *AkhR*. TRiPI On refers to TRiPI activity in response to pulse induction at 29 °C was switched on in adult flies (red bar). TRiPI Off refers to TRiPI activity was at off state in adult flies as they were kept at 18 °C (light red bar). TRiPI On control refers to flies containing the RNAi transgene only, which were also transiently shifted to 29 °C (deep green bar); TRiPI Off control refers to flies of the same genotype compared to TRiPI On control flies, kept constantly at 18 °C (light green). (**C**) Abdominal *GFP* mRNA expression of TRiPI flies containing temperature-sensitive *GFP* transgene displayed substantial higher expression level at day 1 compared to day 10 after *Stim*-TRiPI On as compared to *Stim*-TRiPI Off males. (**D**) High abundance of *AkhR* RNAi effector (siRNA) produced from the *AkhR*-shRNAi transgene at day 0 and maintained at significant high levels at day 10 in the abdomen of *AkhR-*TRiPI On males when compared to *AkhR-*TRiPI Off males. Relative (Rel.) mRNA or siRNA levels are represented as fold change, which refers to the basis value (=1) obtained from corresponding TRiPI Off. (**E**) Fly abdominal *AkhR* mRNA expression level reduced at day 1  and day 10 after *AkhR*- TRiPI On as compared to *AkhR*-TRiPI Off males. Relative (Rel.) *GFP*, siRNA or *AkhR* RNA levels are represented as fold change, which refers to the basis value (=1) obtained from corresponding TRiPI Off flies. Data are presented as means ± standard deviations from 3–6 replicates. All data were analyzed by the two-tailed unpaired Student’s t-test. No *p ≥ 0.05, *p < 0.05, **p < 0.01, ***p < 0.001.

To generate RiPI in adult *Drosophila* flies (Fig. 1A), we employed the GAL4 system^29^ combined with the temperature-sensitive TARGET system^30^ to control UAS-RNAi transgenes in a switchable fat body-specific manner (ts-FB-GAL4)^31^. As a proof of concept, we targeted the Adipokinetic hormone receptor (AkhR) gene, a well-characterized key regulator of lipid mobilization in the fat body^30^. Adult flies at the age of six days, which carried the ts-FB-GAL4 and short hairpin (sh) *AkhR* RNAi transgene (UAS-sh*AkhRi*), were either subjected to a TARGET system-based RiPI (TRiPI) (34–48 hours at the permissive temperature 29 °C; *AkhR*-TRiPI On) or kept continuously at the restrictive temperature 18 °C (*AkhR*-TRiPI Off) (Fig. 1B). To exclude possible confounding effects of the temperature shift per se^32^, we also examined control flies carrying the UAS-sh*AkhRi* transgene only under the two temperature regimes described above (*AkhR*-TRiPI On control and *AkhR*-TRiPI Off control) (Fig. 1B). To demonstrate that our conditional expression system is on-off switchable, we took advantage of ts-FB-GAL4 flies carrying a UAS-GFP reporter transgene (see Material & Methods). As expected, GFP mRNA was significantly up-regulated after the induction pulse (day 1) but was at control levels at day 10 after return to the restrictive conditions (Fig. 1C). Similarly, high levels of *AkhR* siRNA were detected right after the *AkhR*-TRiPI (21 fold higher than that of *AkhR*-TRiPI Off flies at day 0). Strikingly, however, *AkhR* siRNA levels still remained 13-fold higher compared to controls even 10 days after transgene switch-off (Fig. 1D). Consistently, at day 10 after the return to restrictive conditions, *AkhR* mRNA abundance was just around half compared to control flies; a similar gene knockdown level as observed immediately after the end of TRiPI (Fig. 1E). This chronic *AkhR* knockdown in the adipose tissue causes persistent body fat increase (Fig. S1A), which is characteristic for *AkhR* loss-of-function flies^33^. In contrast, we detected no body fat content increase in response to the same temperature shift regimen when ts-FB-GAL4 was absent (Fig. S1A). Importantly, body fat control by tissue-specific, chronic *AkhR*-TRiPI is not restricted to the UAS-sh*AkhR* transgene but also works in combination with a UAS-long double-stranded (ds)*AkhR* transgene (Fig. S1B). Collectively, these data suggest that the turnover of shRNA or dsRNA transgene-derived siRNAs targeting *AkhR* is slow enough to ensure chronic *AkhR* knockdown for at least three weeks after the induction pulse. This finding opens the possibility to monitor chronic disease progression in response to tissue-specific gene interference in an otherwise undisturbed *in vivo* setup.

**Figure 2:**
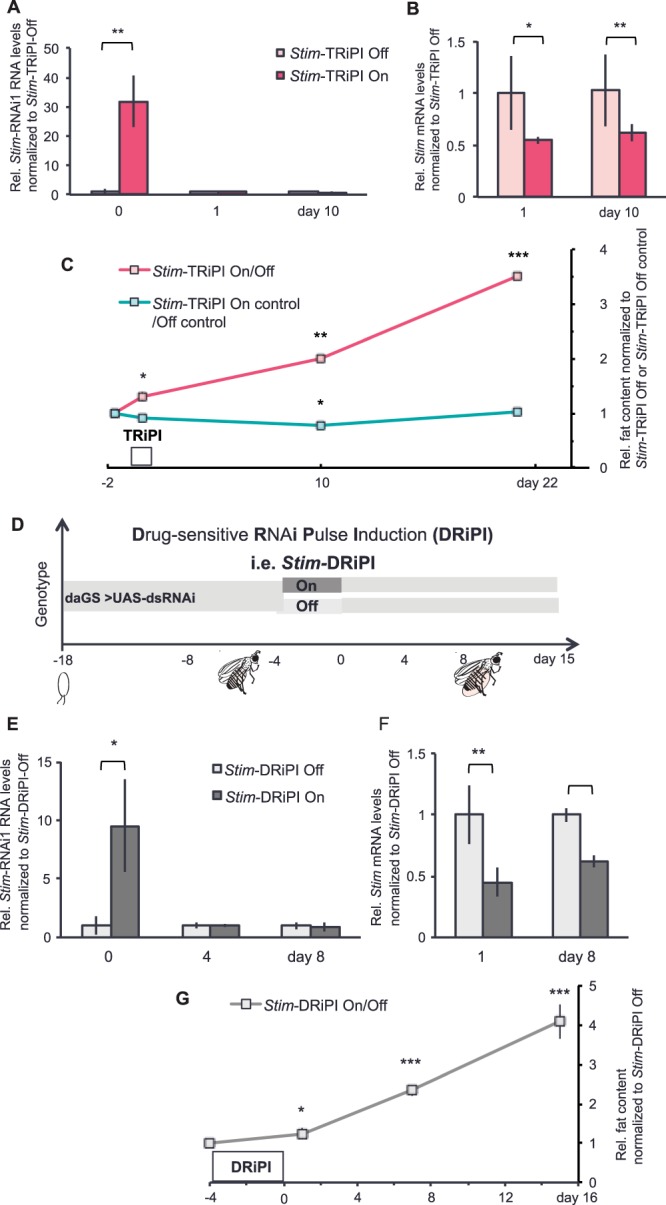
*Stim-*RiPI in adult flies causes chronic impairment of Stim function to promote progressive fly obesity. (**A**) The RNA of *Stim* RNAi1 transgene was effectively overexpressed at day 0, and reduced at day 1 and day 10 after *Stim*-TRiPI On (deep red) as compared to *Stim*-TRiPI Off flies (light red). (**B**) The relative *Stim* mRNA expression level is significantly lower in *Stim*-TRiPI On adult male fly fat body tissue at day 1 and 10 than in *Stim*-TRiPI Off adult males. (**C**) *Stim*-TRiPI On causes a long-term body fat storage increase in adult male flies. (**D**) Detailed scheme showing the time period (4 days, starting at day 4 after the eclosion of adult male flies kept at 25 °C) of Drug-sensitive **RiPI** (**DRiPI**) on targeted genes, i.e. *Stim*. DRiPI On refers to drug-sensitive RNAi transgenic flies, whose RNAi expression was switched on by pulsed drug-feeding (dark grey bar). DRiPI Off refers to drug-sensitive RNAi transgene encoding flies, which were kept under non-induced conditions (light grey bar). (**E**) The dsRNA of *Stim* RNAi1 transgene was efficiently expressed at day 0 after the *Stim*-DRiPI On in adult males, and returned to non-induced levels at days 4 and 8 as compared to *Stim*-DRiPI Off flies. (**F**) The *Stim* mRNA expression level was reduced in adult male fat body tissue at day 1 and 10 after *Stim*-DRiPI On as compared to *Stim*-DRiPI Off flies. (**G**) *Stim*-DRiPI On causes chronic body fat storage increase in adult males. Relative (Rel.) RNA levels or fat content are represented as fold change normalized to the value of *Stim*-TRiPI Off or *Stim*-DRiPI Off flies. Data are presented as means ± standard deviations of 3–6 replicates. All data were analyzed by the two-tailed unpaired Student’s t-test. No *p ≥ 0.05, *p < 0.05, **p < 0.01, ***p < 0.001. Except stated differently, *Stim* RNAi1 transgene line was used in this figure and the following figures.

Next, we targeted by RiPI the ER calcium sensor *Stromal interaction molecule (Stim)*, a key component of the store-operated calcium entry (SOCE). *Stim* is a broadly expressed, essential gene in flies^28^ and mice^26^ and accordingly not accessible to conventional long-term knockout mutant analysis in adult animals. We have previously shown that short-term *Stim* knockdown in the fat body impairs gene function on the mRNA and protein level, which causes excessive lipid accumulation in fly fat cells^15^. Now we subjected six days old adult male flies to an adipose tissue-targeted *Stim*-TRiPI regimen (Fig. 1B**)** using a UAS-ds*Stim* RNAi transgene (*Stim*-RNAi1; Fig. S2A). Consistent with our finding on *AkhR*, the expression of *Stim* dsRNA was effectively switched on and off in response to the *Stim*-TRiPI regimen. *Stim* dsRNA went high immediately after the induction pulse (day 0) but was back to control levels at day 1 and day 10 after the return to the restrictive temperature (Fig. 2A**)**. In contrast to the transient dsRNA pulse, the *Stim* mRNA abundance remained chronically decreased to about 50% at days 1 and 10 (Fig. 2B**)**. In line with the chronic impairment of *Stim* function, *Stim*-TRiPI On flies accumulated significant body fat at day 1 after RiPI, and then progressively added fat (doubling at day 10) to reach remarkable 3.5 times the body fat content of *Stim*-TRiPI Off control flies at day 21 (Fig. 2C**)**. This massive fly obesity development is a universal response to chronic *Stim* impairment, which is largely independent from fly age and sex. Next to young (Fig. 2C) and older (Fig. S2F) unmated male flies, also virgin female flies (Fig. S2C,G) and mated male flies of different ages (Fig. S2B,H) show significant body fat increase three weeks after *Stim-*TRiPI. By contrast, continuously mated female flies of different ages were resistant to *Stim*-TRiPI-dependent obesity (Fig. S2E,I) for currently unknown reasons.

To exclude RNAi off-target effects in the etiology of *Stim*-TRiPI-dependent obesity, we used a second RNAi transgenic line (*Stim*-RNAi2), which targets an independent sequence of *Stim* (Fig. S2A). Consistent with the previous results, the body fat content of *Stim*-RNAi2 flies increased significantly at day 11 after *Stim*-TRiPI and remained higher than controls at day 21 (Fig. S3A). Finally, we confirmed the causal role of *Stim*-TRiPI in fat accumulation by simultaneous expression of *Stim*-RNAi with either one of the two cDNA-based *Stim* transgenes: the RNAi-sensitive *Stim*-RA or the RNAi-resistant *Stim*-Rm transgene (for details see Material & Methods). Fat body-targeted expression of each of the transgenes causes body fat reduction in flies (Fig. S3B,C), which is characteristic for *Stim* gain-of-function^15,25^. RiPI on flies, which carry the *Stim*-dsRNAi and the RNAi-sensitive cDNA-RA transgenes shows progressive fat accumulation as early as day 0 after the pulse induction (Fig. S3D). This fat accumulation profile is consistent with the interpretation that *Stim*-dsRNA downregulates both, the endogenous and the transgenic overexpression of *Stim*. In contrast, concomitant pulse induction of *Stim*-dsRNAi and the RNAi-resistant *Stim*-Rm transgene rescues the normal body fat phenotype at day 0 but not at day 11 or 21 (Fig. S3E). Again, these data are in agreement with the *Stim*-Rm expression being switched off after RiPI, while the dsRNA-mediated *Stim* gene knockdown persists.

To test the general applicability of the RiPI approach for chronic disease modeling, we tested a TARGET-independent switchable gene expression system. Accordingly, we combined *Stim*-dsRNA with the drug (Mifepristone)-inducible geneSWITCH (GS) system^34^ to achieve switchable ubiquitous expression control using *dautherless*GS (*da*GS^35^) (Fig. 2D). As in the case of *Stim*-TRiPI, drug-dependent RNAi pulse induction (DRiPI) of *Stim* caused a significant transient induction of Stim-RNAi just after RiPI (day 0), which returned to control levels at day 4 and following (Fig. 2E). Comparing to fat body-targeted *Stim*-TRiPI, ubiquitous *Stim*-DRiPI also reduced *Stim* mRNA to about 40% of the abundance in control flies at day 8 (Fig. 2F). Consequently, this chronic *Stim* knockdown caused progressive body fat accumulation (Fig. 2G). Notably, this obesity is not only independent from age and sex of the flies but also from food composition. While the absolute fly body fat content varies widely in response to dietary composition, the relative body fat content of *Stim*-DRiPI flies is consistently at least doubled compared to controls on rich and poor food (Fig. S3F).

Collectively, our data present RiPI as a versatile tool to monitor chronic disease progression caused by long-term tissue-specific gene impairment. Specifically, our findings establish *Stim*-RiPI as potent method to study the pathophysiological consequences of chronic SOCE impairment in the fly adipose tissue.

### Chronic adipose tissue impairment of *Stromal interaction molecule* causes metabolic disease in flies

**Figure 3:**
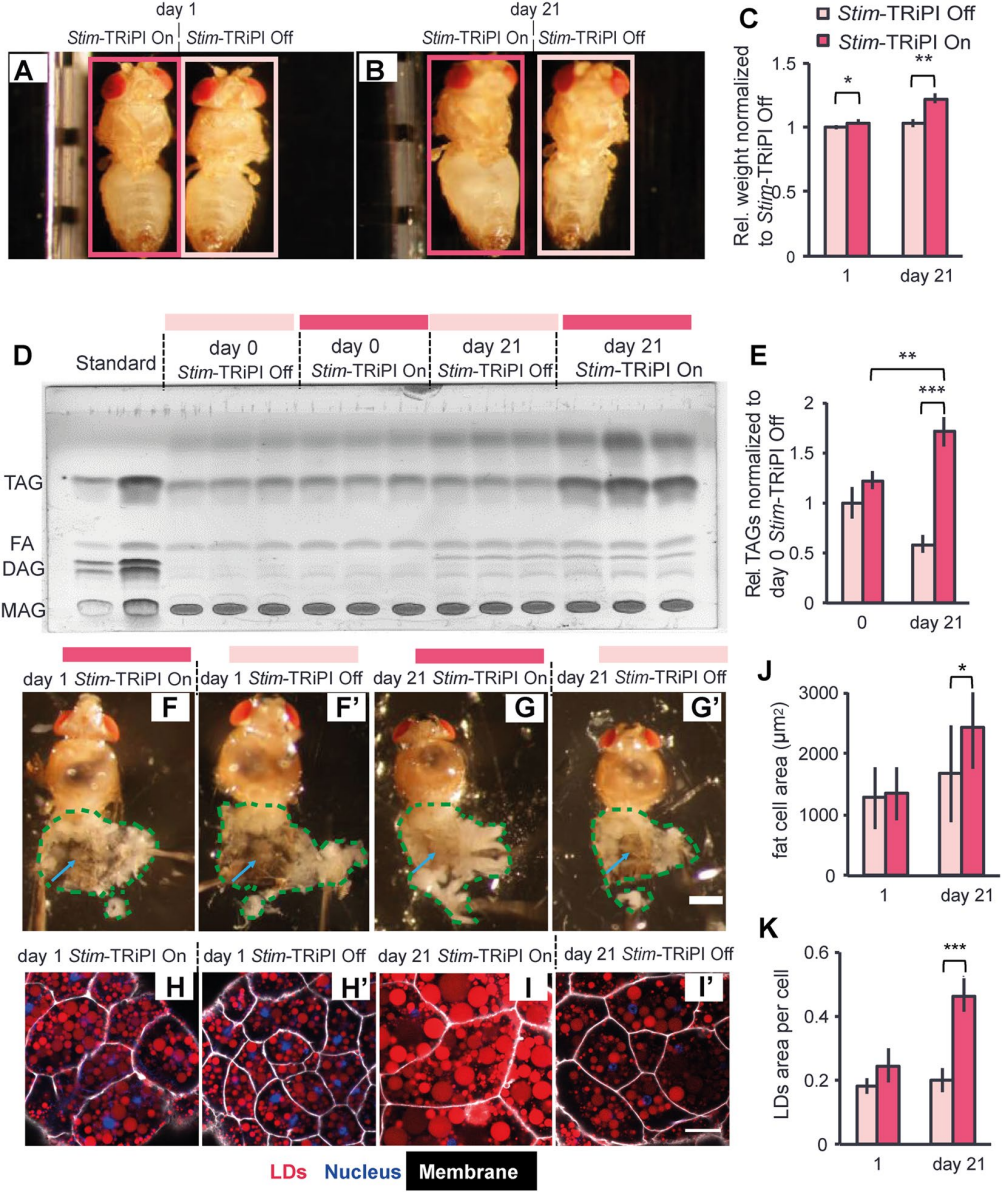
Chronic *Stim* dysfunction causes fat body cell hypertrophy and obesity in *Drosophila*. Images of day 1 (**A**) and days 21 (**B**) *Stim*-TRiPI flies display the expanded abdomen developed at day 21 of *Stim*-TRiPI On (encircled by red rectangle) as compared to *Stim*-TRiPI Off (encircled by light red rectangle) adult male flies. (**C**) Increased wet body weight of day 21 Stim-TRiPI On adult male flies as compared to *Stim*-TRiPI Off flies. (**D**) Major neutral lipid classes at day 0 and day 21 of *Stim*-TRiPI On and the *Stim*-TRiPI Off adult male flies, separated by thin layer chromatography illustrates specific accumulation of storage fat (triacylglycerol; TAG); MAG: Monoacylglycerol; DAG: Diacylglycerol; FA: Fatty acids; Standard refers to a defined mixture of FA, MAG, DAG, and TAG. (**E**) Integrated intensity quantification of the TAG bands in (**D**). Dissected flies show similar morphology of fat body tissue (white tissues encircled by green stippled lines) at day 1 but more fat tissue (the area is highlighted by blue arrow) at day 21 of *Stim*-TRiPI On (**F**,**G**; under the red line) as compared to age-matched *Stim*-TRiPI Off (**F’**,**G’**; under the light red line) adult male flies. Scale bar in F, F’, G, G’ represents 0.5 mm. Visualization of lipid droplets (stained by LD540; red), cell membranes (stained by Cell Mask Deep red; white) and nuclei (stained by DAPI; blue) in abdominal fat body tissue of adult male flies at day 1 (**H**) and day 21 of *Stim*-TRiPI On (**I**) and the corresponding control male (*Stim*-TRiPI Off) flies at day 1 (**H’**) and day 21 (**I’**) shows an increased size of fat body cells (**J**) and increased lipid droplets (LD) area in fat cells (**K**) at day 21. Scale bars in H, H’, I and I’ represent 50 μm. Data are presented as means ± standard deviations from 3–6 replicates. All data were analyzed by the two-tailed unpaired Student’s t-test. No. *p ≥ 0.05, *p < 0.05, **p < 0.01, ***p < 0.001.

After having demonstrated that *Stim*-RiPI flies suffer from excessive fat accumulation, we asked whether these flies also display other phenotypes characteristic for mammalian obesity such as increased girth, elevated body weight, and reduced physical fitness. While *Stim*-TRiPI On and *Stim*-TRiPI Off flies are indistinguishable at day 1 (Fig. 3A), abdominal girth of day 21 *Stim*-TRiPI On flies is visibly inflated compared to *Stim*-TRiPI Off flies (Fig. 3B). Consistently, *Stim*-TRiPI On flies gained about 22% body weight in three weeks to become 18% heavier than age-matched *Stim*-TRiPI Off flies (Fig. 3C) and a dry weight gain of *Stim*-TRiPI On flies is also observed (Fig. S3G). Consistent with inflated abdominal girth, and increased body weight, thin layer chromatography (TLC) data confirmed that the body TAG content of day 21 Stim-TRiPI On flies was 90% higher compared with day 21 Stim-TRiPI Off flies (Fig. 3D,E). Fly fat body tissue is functionally similar to mammalian adipose tissue and liver, which is a major organ for fat and glycogen reserve^12^. To check if the fly adipose tissue increases, we examined fly fat body tissues in dissected abdomen and found more expanded fat tissue in day 21 *Stim*-TRiPI On flies than day 1 Stim-TRiPI On and Off as well as  day 21 *Stim*-TRiPI Off flies (Fig. 3F,F‘, G,G‘). At the cellular level, fat body tissue *ex vivo* staining showed that fat cell size is unaltered in day 1 *Stim*-TRiPI On flies (Fig. 3H,H’,J), while it increased by around 46% at day 21 (Fig. 3I,I’,J). In addition, the cumulative LD area per fat body cell of *Stim*-TRiPI On flies was around 2.3 fold higher compared to day 21 *Stim*-TRiPI Off flies (Fig. 3I,I’,K). These data indicate that the chronic dysfunction of *Stim* causes severe fly fat body cell hypertrophy.

Consistent with their increased fat reserves, obese *Stim*-TRiPI flies are more starvation resistant compared with control flies (Fig. S4A**)**. Noteworthy, substantial *post mortem* lipid stores suggest a lipid mobilization impairment in *Stim*-TRiPI flies **(**Fig. S4B). Much in contrast to fat, the glycogen storage under *ad libitum* feeding and the glycogen mobilization upon starvation is comparable between *Stim*-TRiPI On and *Stim*-TRiPI Off control flies (Fig. S4C). By contrast, we found that day 10 *Stim*-DRiPI On flies displayed substantially higher level (around 1.9 fold) of circulating sugar levels compared to the value of day 10 *Stim*-TRiPI Off control flies (Fig. S4D). These results demonstrate that the metabolic dysregulation of body energy stores includes not only a defect in TAG mobilization but also hyperglycemia, which likely contributes to or at least correlates with the obesity phenotype.

We asked next, whether *Stim*-TRiPI-dependent obesity in flies correlates with physical fitness impairment. Indeed, the climbing ability of day 24 *Stim*-TRiPI flies was reduced by 40% compared to controls (Fig. S4D). Also, the median lifespan of *Stim*-DRiPI flies was significantly reduced by about 21% compared to corresponding control flies (Fig. S4E), which is reminiscent of the increased mortality correlated with human obesity^4^. Collectively, these data show that the *Stim*-TRiPI model specifically affects the storage lipid metabolism and recapitulates hallmarks of mammalian obesity.

**Figure 4:**
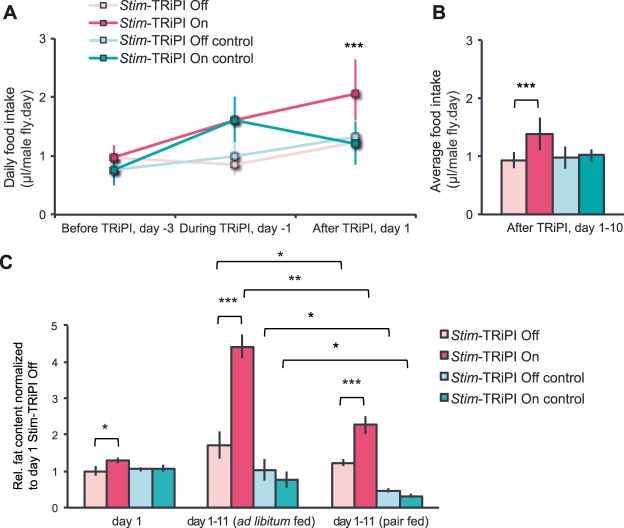
Hyperphagia contributes to obesity in *Stim*-TRiPI in adult male flies. (**A**) *Stim*-TRiPI On results in hyperphagia of male flies from *Stim*-TRiPI On induction onwards compared to *Stim*-TRiPI Off flies. Note that *Stim*-TRiPI On control adult male flies also showed a transient increase of food intake during the induction period, which returned to the same level as the corresponding *Stim*-TRiPI Off control flies at day 1 (n = 34–48). (**B**) Average daily food intake increases over a period of 10 days of Stim-TRiPI On compared to the *Stim*-TRiPI Off flies. (**C**) Pair-feeding reduces but not fully suppresses obesity development in *Stim*-TRiPI On male flies over a period of 11 days when compared to *ad libitum* fed flies (n = 4–8). Relative (Rel.) fat content is represented as fold change, which refers to the basis value (=1) obtained from corresponding *Stim*-TRiPI Off flies. Data are presented as means ± standard deviations. All data were analyzed by the two-tailed unpaired Student’s t-test. No *p ≥ 0.05, *p < 0.05, **p < 0.01, ***p < 0.001.

Next, we asked how long-term knockdown of *Stim* changes systemic energy homeostasis. Consistent with previous acute RNAi expression studies^15^, we observed that *Stim*-TRiPI On flies increased food intake by 90% immediately after pulse induction (Fig. 4A) and these flies stayed hyperphagic the following 10 days (Fig. 4B). Similarly, food intake was increased by around 50% in *Stim*-DRiPI On flies for eight days using this alternative RNAi induction system (Fig. S5A). These data suggest that hyperphagia contributes to extra fat accumulation. In line with this hypothesis are the results from restrictive pair-feeding experiments. In this paradigm, *Stim*-TRiPI On flies were offered slightly less than the food amount consumed by *ad libitum* fed *Stim*-TRIP Off flies to largely match the dietary intake of experimental and control flies. Under pair-feeding, the body fat content of *Stim*- TRiPI On flies increased by moderate 127% during the first ten days, and reach 1.85-fold the value of age-matched *Stim*-TRiPI Off flies (Fig. 4C). The corresponding body fat values increase under *ad libitum* fed conditions are 343% during the first ten days (with a 30% increase already at day 1 after pulse induction) to reach 2.57-fold the value of *Stim*-TRiPI Off flies (Fig. 4C). To assess the contribution of hyperphagia-dependent lipogenesis we used dietary ^14^C glucose pulse-labeling to compare *de novo* lipid synthesis between *Stim*-DRiPI On and Off flies (Fig. S5B) as glucose intake is known to drive lipogenesis^36^. Indeed, the 24 h ^14^C incorporation into neutral lipids of *Stim*-DRiPI On flies was almost five times higher compared to *Stim*-DRiPI Off flies (Fig. S5C), which substantially exceeds the corresponding incorporation into polar lipids (79%; Fig. S5D) or the food intake increase. These results suggest that in addition to hyperphagia, hyperactivity of TAG biosynthesis and/or reduced energy expenditure causes the excessive fat accumulation. Therefore, we used the respirometry-based metabolic rate estimation in fly^37^ to detect a 29% decrease in CO_2_ production at noon and a 17% decrease in the evening in the of *Stim*-DRiPI On flies compared to the corresponding control flies (Fig. S5E). Collectively, these data demonstrate that hyperphagia, increased lipogenesis, and reduced energy expenditure contribute to fly obesity in response to chronic *Stim* knockdown by unknown molecular mechanisms.

### Adipose tissue lipolysis dysfunction, Adipokinetic hormone resistance, and insulin signaling impairment caused by chronic impairment of *Stromal interaction molecule*

**Figure 5:**
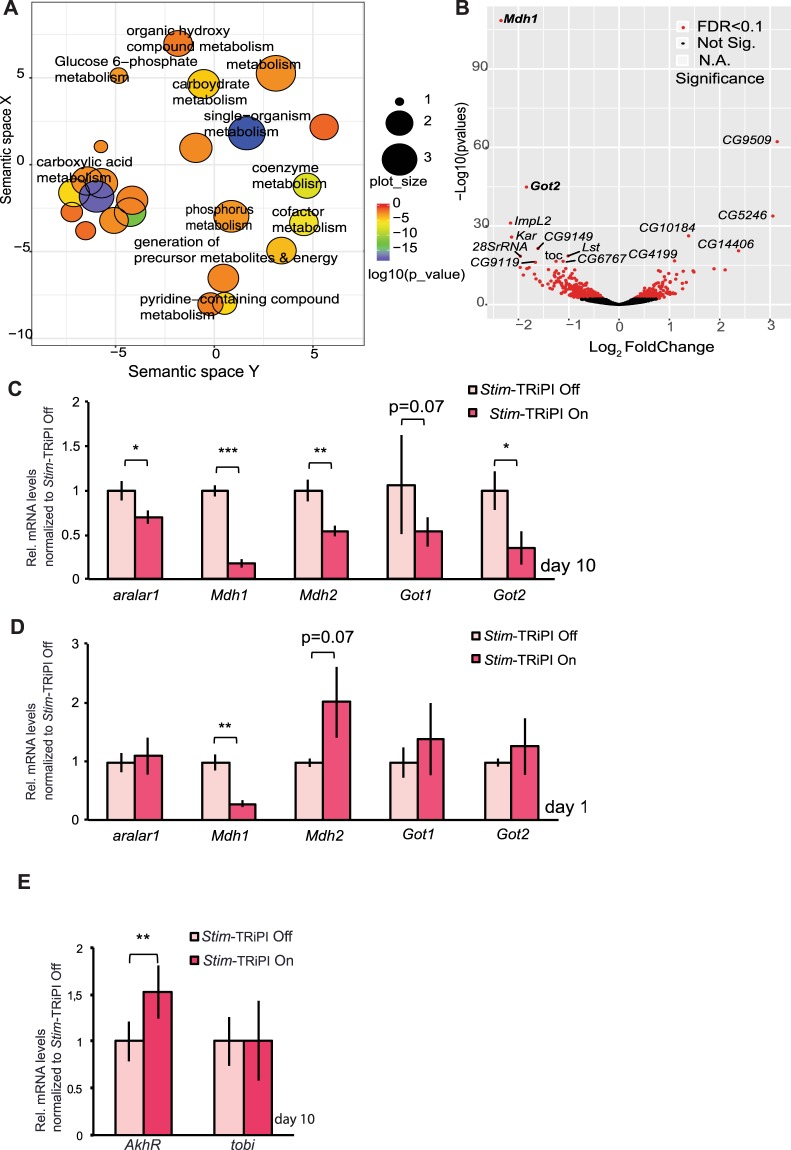
Fat body transcriptome profile changes indicate impairment of Akh signaling in response to chronic *Stim* dysfunction. (**A**) Biological process Gene Ontology term analysis of genes down-regulated in response to chronic *Stim* impairment in the fat body results in enrichment of genes involved in metabolic processes such as carbohydrate, carboxylic acid, coenzyme, carboxylic acid, coenzyme metabolism, cofactor metabolism, Glucose 6-phosphate, organic hydroxyl compound, pyridine-containing compound and phosphorus metabolic processes, generation of precursor metabolites & energy. For details see supplementary information. (**B**) Volcano plot illustration of genes transcriptionally responsive to chronic *Stim* dysfunction, which were selected on the basis of their differential expression (log2-fold change >0.5 or <−0.5) and p value adjusted with false discovering rate (FDR; value < 0.1) according to RNAseq data. Gene symbols of 15 most differentially regulated genes are labeled. Among them, the two repressed genes with highest confidence (*Mdh1, Got2)* were labelled in bold. (**C**) RT-qPCR confirms transcriptional down-regulation of the malate-aspartate shuttle genes *aralar1, Mdh1, Mdh2, Got1 and Got2* at day 10 after *Stim*-TRiPI On as compared to *Stim*-TRIP Off flies. (**D**) Among the malate-aspartate shuttle genes only *Mdh1* is an early response gene to *Stim*-TRiPI On as revealed by comparison to the *Stim*- TRiPI Off flies. (**E**) RT-qPCR identifies transcriptional up-regulation of the *AkhR* gene but no change on *tobi* mRNA level. Data are presented as means ± standard deviations from 3–6 replicates. All qRT-PCR data were analyzed by the two-tailed unpaired Student’s t-test. No *p ≥ 0.05, *p < 0.05, **p < 0.01, ***p < 0.001.

To address the tissue-autonomous mechanisms of chronic *Stim* knockdown, we performed differential transcriptome profiling by comparative RNAseq analysis on dissected fat body tissues of day 10 *Stim*-TRiPI On and Off flies (Fig. S6A–C). Unsurprisingly, gene ontology analysis revealed a complex regulatory response to chronic SOCE impairment suggesting the down-regulation of various metabolic processes (Fig. 5A). A group of significantly down-regulated genes comprises *aralar1, Mdh1, Mdh2, Got1*, and *Got2* (Fig. 5B,C), all of which encode enzymes involved in NADH shuttling between mitochondria and cytoplasm (Fig. S6D). To understand the correlation between Akh signaling and genes involved in NADH shuttling, we co-analyzed the RNAseq dataset of fly larval fat body samples in response to Akh overexpression vs. control condition^38^ and our RNAseq dataset of adult fly fat body tissue under *Stim*-TRiPI On vs. Off condition. Intriguingly, 47 genes were up-regulated in larval fly fat body overexpressing Akh but down-regulated in *Stim*-TRiPI On adult fly fat body tissue: four of them are *Mdh1, Got1, Got2* and *aralar1*, which could be considered as Akh signaling reporter genes (Fig. S7A). Moreover, *Mdh1* mRNA is down-regulated as early as day 1 in *Stim*-TRiPI On flies (Fig. 5D) but not in *Stim*-TRiPI On control flies (Fig. S7B). Another gene contrarily expressed under the described conditions is *Gprk2* (Fig. S7A), which is directly correlated with cAMP levels in fly tissue^39^. Consistently, both RNAseq and RT-qPCR show that the *AkhR* mRNA level is up-regulated in the fat body of *Stim*-TRiPI On flies, however not the AkhR downstream signaling gene *tobi*^40^ (Fig. 5E). Collectively, these finding suggest a differential impairment of Akh signaling in the fat body of obese *Stim*-TRiPI flies.

Previous research showed that Akh/AkhR signaling is causative for lipid mobilization^33,41^. Consistently, RNAseq indicates the transcriptional down-regulation of the paralogous lipase genes, *doppelgänger of brummer (dob)* and *brummer (bmm)*, which encodes the fly homolog of mammalian adipose triglyceride lipase (ATGL)^42^ in response to chronic *Stim* impairment (Fig. S8A). Moreover, this analysis suggests a coordinated down-regulation of mitochondrial and peroxisomal β-oxidation pathways in obese *Stim*-TRiPI On flies (Fig. S8A). We confirmed the transcriptional down-regulation of lipid catabolic pathway genes by selected RT-qPCR analyses of *bmm*, *carnitine O-palmitoyltransferase* (*CPT1)*, *dACADVL*, and *dATP5B* (Fig. S8A,C). In contrast, *midway (mdy)*, which encodes the homolog of the key human lipid synthesis enzyme diacylglycerol acyltransferase 1 (DGAT1), was only significantly up-regulated at day 1 but not at day 10 in *Stim*-TRiPI On fat body (Fig. S8D). Similarly, other key genes involved in lipid biosynthesis such as *dFAS* (fatty acid synthase gene), *dACS* (Acetyl Coenzyme A synthetase gene), *dLipin* (Mg^2 +^ -dependent PA phosphatase gene) were not significantly up-regulated at day 10 in *Stim*-TRiPI On fat body (Fig. S8D,E). Collectively, these data suggest that a partial Akh resistance as well as impairment of lipolysis and mitochondrial function mediate obesity progression in the fat body in response to chronic *Stim* knockdown.

To understand why *Stim*-DRiPI On flies have higher circulating sugar levels, we checked the expression of the Hex-C gene, a homolog of mammalian *Glucokinase* (*GCK*) likely involved in glucose clearance by fly fat body^43^ and insulin signaling^44^. *Stim*-TRiPI On flies show around 35% reduced expression of the Hex-C gene at day 10 (Fig. S9A), which might account for the hyperglycemia. Since insulin-deficient flies display hyperglycemia^45^ and insulin signaling in the fat body promotes circulating sugar clearance^46^, we checked peripheral insulin signaling in *Stim*-TRiPI On flies. The insulin signaling target gene *d4EBP*’s expression was not significantly up-regulated at day 1, and mildly but significantly up-reguated by around 25% at day 10 (Fig. S9B), which is repressed by the insulin signaling through active phosphorylated Akt^47^. Besides, although insulin signaling was shown to promote fly fat cell proliferation and fat accumulation^48^, we did not find a measurable increase of new DNA synthesis indicated by EdU staining in *Stim*-TRiPI On fly fat body tissues (Fig. S9C). Consistently, the phosphorylated Akt (at Ser505^43^ but not Thr342^49^) level, the downstream readout of insulin signaling^50^, shows a reduced trend by 37% in *Stim*-DRiPI On peripheral tissues (Figs S9D, S10A, S11A,B,C). Moreover, loss-of-function of the three central insulin peptide genes *dIlp2,3,5*, the master regulators of sugar homeostasis^51,52^, did not prevent the extra fat accumulation of *Stim*-DRiPI On flies (Fig. S9E). Consistent with our previous finding^15^, insulin producing cells (IPCs) of *Stim*-TRiPI On flies also show reduced staining intensity of dIlp-2 by around 30% (Fig. S9F,G), indicative of increased dIlp-2 secretion^53^. In line with this, RNAseq and RT-qPCR analysis identified the up-regulation of insulin secretion promoting gene *CCHamide-2* (*CCHa2*)^54^ by around 67% and *dawdle (daw)*^55^ by around 35%, but down-regulation of insulin signaling inhibiting gene *Ecdysone-inducible gene L2* (*ImpL2*)^56^ by around 85% and insulin secretion suppression gene *Limostatin* (*Lst*)^57^ by around 70% at the mRNA level (Fig. S10A). To measure circulating dIlp-2 directly, we combined the *Stim*-DRiPI system with dIlp-2HF (epitope-tagged *dIlp-2* gene)^52^ and found no significant difference on dIlp-2HF levels between obese *Stim*-DRiPI On and *Stim*-DRiPI Off flies (Fig. S10D). While the contradictory findings on circulating dilp levels need future research attention, the results collectively show that insulin signaling is impaired in the fat body tissues of obese *Stim*-RiPI flies and partially dispensible for long-term body fat storage increase.

### Systemic Adipokinetic hormone signaling controls hyperphagia in response to chronic *Stromal interaction molecule* dysfunction in the fat body

**Figure 6:**
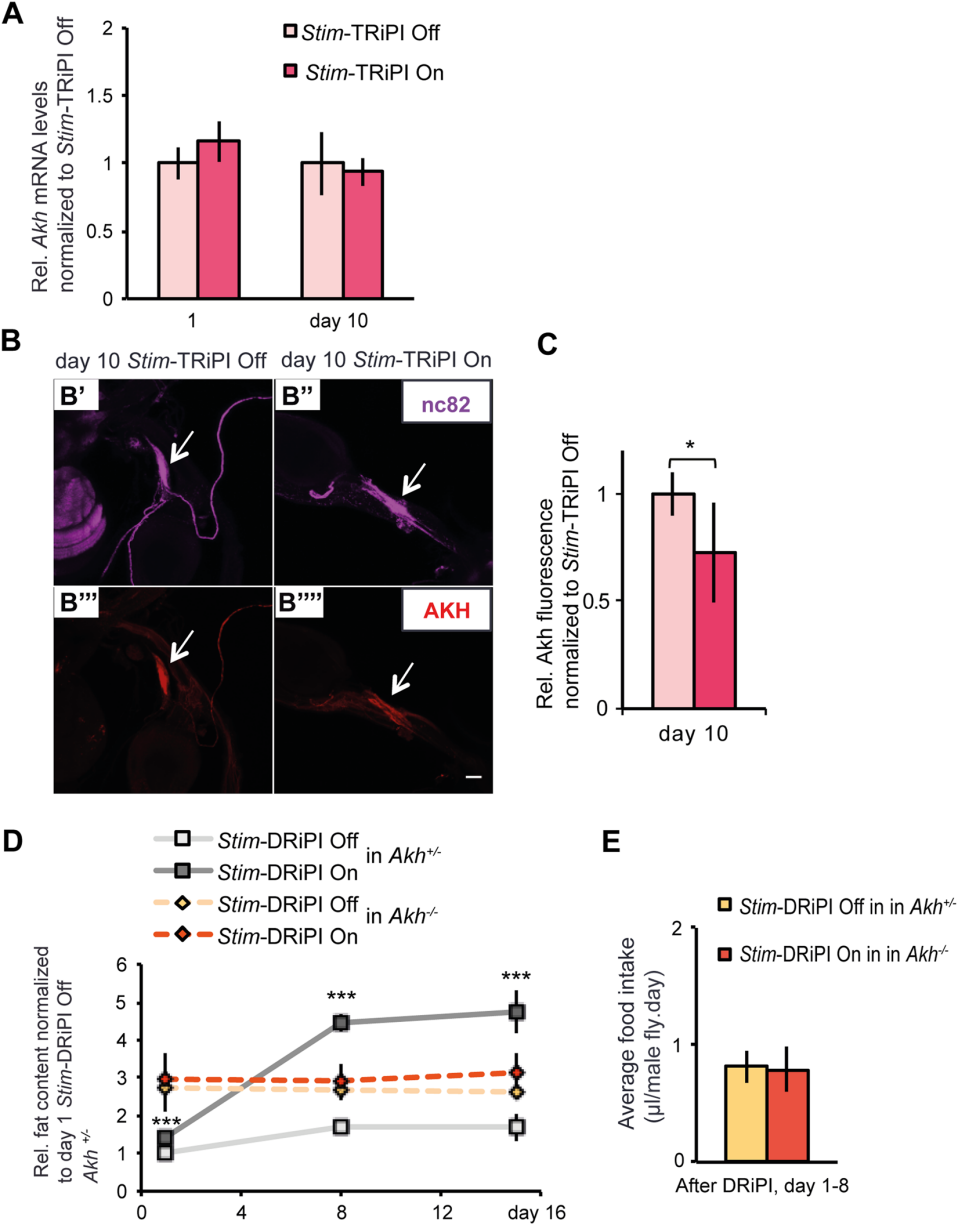
*Stim* dysfunction in the fat body exerts systemic control of hyperphagia and obesity possibly via Akh secretion from neuroendocrine cells. (**A**) *Akh* mRNA expression levels are unresponsive to *Stim*-TRiPI On as compared to *Stim*-TRiPI Off flies in adult male flies at day 1 and 10 after RNAi induction (n = 3–6). (**B**) Reduced Akh peptide storage in corpora cardiaca cells (red) visualized by immunohistochemistry on adult male flies at day 10 under control condition (*Stim*-TRiPI Off; B”’) or at day 10 after *Stim*-TRiPI On (B””). Note that anti-Bruchpilot antibody NC82 was used to counterstain the corpora cardiaca cells (B’ and B”). Scale bars in B represent 50 μm. (**C**) Quantification of Akh reduction in corpora cardiaca cells of day 10 *Stim*- TRiPI On adult male flies as compared to corresponding control adult male *Stim*-TRiPI Off flies (n = 7–12). (**D**) *Stim*-DRiPI On causes progressive obesity in heterozygous but not in homozygous *Akh* mutant males. Relative (rel.) fat content is represented as fold change normalized with respect to the value of day 1 *Stim-*DRiPI Off flies in *Akh* heterozygous (n = 6). (**E**) Loss-of-Akh suppresses the hyperphagia of *Stim*-DRiPI On male flies. No difference between average daily food intake from day 1 to day 8 in *Akh* mutant male flies with (*Stim*-DRiPI Off) or without (*Stim*-DRiPI Off) *Stim* dysfunction in the fat body (n = 32). Data are presented as means ± standard deviations. All data were analyzed by the two-tailed unpaired Student’s t-test. No *p ≥ 0.05, *p < 0.05, **p < 0.01, ***p < 0.001.

Adipokinetic hormone signaling does not only promote lipid catabolism in the fat body, but also regulates food intake with ectopic Akh expression causing hyperphagia^58,59^. Therefore, we asked if *Stim* dysfunction of fat body tissue promotes food intake via Akh signaling. We first quantified the *Akh* mRNA levels of day 1 and day 10 *Stim*-TRiPI On flies to find no expression differences compared to control flies (Fig. 6A). Since Akh signaling is controlled by Akh peptide secretion from the neuroendocrine corpora cardiaca (CC) cells^60^, reduced Akh immunostaining intensity in CC cells is used as a proxy for increased secretion of the hormone^60,61^. Therefore, we assessed Akh peptide levels by comparative immunohistochemistry on dissected CC cells of day 10 *Stim*-TRiPI On/Off flies using antibodies directed against Akh and the synaptic cytoskeletal protein Bruchpilot (served as neuronal marker for signal normalization) (Fig. 6B,C). Normalized Akh staining intensity in CC cells of *Stim*-TRiPI On flies was significantly reduced by around 27% compared to *Stim*-TRiPI Off flies (Fig. 6B,C) suggesting increased Akh secretion in response to chronic SOCE impairment in the fat body. To directly address the role of Akh in obese flies subject to chronic *Stim* fat body dysfunction, we compared the body fat content and the food intake of *Akh* heterozygous and homozygous mutant flies subjected to *Stim*-DRiPI On/Off (Fig. 6D,E). Loss-of-Akh completely suppressed obesity development (Fig. 6D) and hyperphagia (Fig. 6E) in *Stim*-DRiPI On flies.

Since *Stim*-TRiPI On flies have higher circulating sugar levels than control flies, the increased Akh secretion cannot be triggered by lower circulating sugar levels as reported previously^60^. Thus, we propose that increased Akh secretion in *Stim*-RiPI On flies might be regulated by unknown fat body tissue secreted factors. RNAseq analysis on *Stim*-TRiPI fat body tissues identified 16 differentially expressed genes encoding proteins belong to “secreted” group (supplementary data file 2). Interestingly, *Lst, CCHa2, daw, Est-6, and PGRP-SD* were also transcriptionally regulated in fly larval fat body overexpressing Akh (Fig. S10B), which qualifies them as fat body-derived signal candidates to regulate Akh secretion. Collectively, these results support the model that the chronic impairment of *Stim* activity in the fat body remotely triggers Akh secretion from the CC cells by currently unknown mechanisms, which in turn stimulates food intake and thereby contributes to obesity progression.

## Discussion

### Chronic knockdown of target genes by RNAi Pulse Induction (RiPI)

Here we present *in vivo* evidence for chronic targeted gene knockdown following RiPI in the adult *Drosophila* fat body. We found that a short pulse induction of shRNA targeting the *AkhR* gene generates persisting siRNAs, which causes significant down-regulation of *AkhR* for at least 10 days. Persistence of RNAi has been associated with RNA-dependent RNA polymerase (RdRP)-mediated siRNA amplification in *C. elegans*^62,63^ and in human cells^64^. In *Drosophila*, however, it remains controversial whether the genome encodes a functional RdRP^65,66^. Therefore, slow degradation of the transgene-derived siRNAs might confer the chronic gene knockdown. In fact, RNAi effector double-stranded siRNAs (21nt and 24nt) are more stable than the 18nt double-stranded RNAs in the human cytosolic extract^67^. Moreover, in human HEK293T cells, the anti-sense strand of siRNA is more resistant to intracellular nucleases compared to the sense strand of the siRNA duplex, which is likely due to the incorporation of anti-sense siRNAs into the activated RNA induced silencing complex (RISC)^68,69^. Therefore, the involvement of RISC might allow the slow degradation of siRNAs in adult *Drosophila* fat body cells. The slow decline of the siRNA level is apparently sufficient to chronically knockdown the endogenous gene expression of *AkhR* and *Stim*, which causes progressive body fat increase. This mode of action is further supported by the fact that pulsed overexpression of RNAi resistant *Stim*-mRNA only transiently rescues the fat content increase due to *Stim*-TRiPI. Consistently, long-term gene silencing (at least 11 days) is also observed in adult flies after injection of low concentrations of dsRNAs^70^. Similarly, in an EGFP-transgenic mouse model, the inhibition of the reporter expression lasts as long as two months after siRNA injection^71^. In summary, we show here that *in vivo* RiPI generates long-lasting RNAi, which allows chronic knockdown of target genes in a tissue-specific manner. We propose that RiPI is a versatile tool to study causative relationships and temporal sequences in inter-organ communication processes.

### A *Drosophila* obesity model triggered by chronic *Stromal interaction molecule* dysfunction in the fly adipose tissue

Using RiPI, we established a *Drosophila* obesity model based on chronic, adipose tissue-directed knockdown of *Stim*, which shares remarkable similarity to characteristics of human obesity. First, the visibly enlarged abdomen of the obese flies corresponds to increased waist circumference, which gains importance as meaningful parameter to assess android adiposity^72^. Similarly, body fat accumulation causes significant weight gain, another readout to quantify obesity in rodents^73^ and human^74^. Second, the excessive fat accumulation correlates with climbing deficits of the obese flies, with physical fitness reduction being another hallmark of human adiposity^75^. Moreover, obese *Stim*-TRiPI flies have reduced life span, which is reminiscent of the higher mortality rates in human obesity patients^76^. Third, we demonstrate that early-onset hyperphagia drives the positive energy balance in *Stim*-TRiPI flies. Consistently, increased food intake is the major driver of human obesity^5^. Hyperphagia is linked to increased dietary glucose conversion into storage fat in obese *Stim*-TRiPI flies. Notably, increased food intake and elevated glucose conversion into storage lipids has also been reported after silencing obesity blocking neurons in the fly central brain^77^. With hyperphagia being an important contributor, obesity development in *Stim*-RiPI flies is not monocausal. It is noteworthy that the rise in fat storage in *Stim*-DRiPI substantially exceeds the food intake increase. Moreover, matching the food intake of *Stim*-TRiPI On and Off flies still results in body fat accumulation. Importantly, there is a significantly reduced metabolic rate of *Stim*-DRiPI flies. Finally, the observed hyperglycemia at day 10, physical fitness reduction at day 24 and shortened life span of *Stim*-TRiPI On flies are associated with obesity development, similar to type 2 diabetes (T2D)^78^, exercise intolerance^79^ and mortality^80^, which are also highly correlated with human obesity. In summary, chronic knockdown of *Stim* in the adult fat body causes fly obesity by a number of physiological factors culminating in organismal energy imbalance similar to mammalian adiposity.

### Chronic knockdown of *Stromal interaction molecule* causes adipose tissue dysfunction

Our study highlights the critical roles played by *Stim* in interaction with Akh/AkhR signaling and insulin signaling in the fly fat body tissue. Reduced expression of *Mdh1* and *Gprk2* suggests impaired Akh/AkhR signaling in the fat body of *Stim*-TRiPI flies. Mammalian MDH1 has been linked to glycolysis in cells with mitochondrial dysfunction^81^, obese *Stim*-TRiPI On flies display normal glycogen storage and mobilization during starvation. Similar findings are also observed in *Akh*^*A*^, *Akh*^*AP*^, and *AkhR*^1^ mutant fly larvae^82^ and adult flies, albeit their capability to mobilize glycogen is weakly impaired^41^. A possible explanation is that flies employ corazonin, a starvation-responsive pathway complementary to Akh, to utilize glycogen^83^. In addition to storage glycogen, the reduced expression of genes involved in lipolysis predicts an impairment of starvation-induced storage lipid mobilization. Indeed, obese *Stim*-TRiPI flies display an abnormal lipid mobilization profile under starvation and die with residual fat resources. Similarly, impaired lipid mobilization is also observed in flies with loss-of-function mutation in the TAG lipase gene *bmm*^42^ or in flies lacking either *InsP3R*^84^ or *AkhR*^33^. Consistently, loss-of-function of STIM1/2 in mammalian cells, also impairs lipolysis via down-regulation of cAMP^26^. Moreover, decreased catecholamine-stimulated lipolysis has been identified in human obese individuals^85^. Collectively, our results show that fat body tissue of obese *Stim*-RiPI On flies is resistant in response to Akh signaling, which drives the obesity development.

Moreover, our study supports the possibility to model T2D in adult flies. Obese *Stim*-TRiPI flies show reduced expression of the glucose clearance gene *Hex-C*, whose mammalian homolog was also suppressed in T2D patients^86^. Besides, we also provide evidences to support that obese *Stim*-TRiPI flies have hyperglycemia, impairment of insulin signaling in fat body tissue, and larger lipid droplets. Similar features were also described in fly larvae reared on high sugar diet^36,87^, which resemble mammalian insulin resistance^88^. Regarding unchanged circulating dIlp-2 level in obese *Stim*-DRiPI flies, insulin-like peptide secretion might be interfered by the knockdown of *Stim* in the insulin producing cells of *Stim*-DRiPI flies mediated by ubiquitous driver daGS, more investigation on circulating insulin levels of obese *Stim*-DRiPI flies by specific driver need to be done in future. Interestingly, the indicators of insulin signaling impairment mentioned above occur at later stage of *Stim*-RiPI obesity development, and accordingly are possibly the consequence of *Stim*-TRiPI On mediated-fat gain, which also supports the concept that obesity compromises insulin signaling.

### Metabolic fat body dysfunction drives organismal energy imbalance

Apart from the specific role of the fat body in storage lipid handling and glucose clearance, we show that chronic knockdown of *Stim* in this organ remotely promotes Akh secretion from the fly CC neuroendocrine cells, which leads to hyperphagia. Our RNAseq and gene expression analysis indicate a list of genes encoding candidate hormone or secreted proteins. Among them, CCHa2, daw, and Lst has been shown to function as hormones to regulate insulin-like peptide secretion^54,55,57^. In addition, CCHa2, daw, Lst are also regulated by Akh overexpression in opposite direction. Whether differential expression of these genes mentioned above mediate the (mis)communication between the fat body and the CC cells is currently unknown. Nevertheless, the communication between the fat body and the CC cells is essential for the food intake increase as well as further obesity development induced by long-term knockdown of *Stim*. Interestingly, a study provided evidence that muscle tissue in flies communicates with the CC cells to control Akh secretion via the myokine Unpaired2 (Upd2)^89^. Upd2 had been previously shown to act as adipokine, which signals the fed state from the fat body. Unlike mammalian leptin, Upd2 remotely acts on insulin-producing cells in the central brain to regulate insulin secretion but not food intake^90^. Recently, *Akh* mRNA expression was shown to be regulated by a gut-neuronal relay via midgut-secreted peptide Buriscon α in response to nutrients^91^. Given the fact that the transcription of *Akh* is unaffected in *Stim*-RiPI On flies, identification of the adipokine, which regulates the Akh release directly or indirectly to affect food intake in the *Stim*-RiPI fly obesity model requires future research efforts.

In conclusion, our work introduces RNAi Pulse Induction as a novel *in vivo* paradigm for chronic, tissue-specific gene interference. RiPI makes essential genes accessible to long-term functional analysis in the adult fly, as exemplified here by establishing a *Drosophila* obesity model caused by chronic knockdown of *Stim* in the adult fat body. Moreover, this study reveals, that the fat body integrates the tissue-autonomous and the systemic branches of Akh signalling: by regulation of lipid mobilization via SOCE in the fat body, and possibly by remote-control of Akh secretion from the CC cells. Recently, the evolutionarily conserved role of SOCE in controlling energy metabolism has attracted the interest of mammalian studies^26,27^. While Akh is structurally not conserved to humans, there is a growing number of remotely-controlled orexigenic peptide hormones in mammals with asprosin being one of the latest additions^92,93^. Collectively, our findings in the fly add further evidence to the existence of conserved regulatory principles in animal energy homeostasis control emanating from SOCE signalling in fat storage tissues.

## Material and Methods

### Fly stocks and husbandry

The following *Drosophila* fly stocks were used in this study: *Stim*RNAi1^94^, ts-FB-Gal4^31^, *da*-GS^35^, *dIlp2*–*3,5 mutant*^51^, *Akh*^*A*^ ^41^, *Ilp2*^*1*^*gd2HF*^52^. Balancer line 2 (#7198), *AkhR* RNAi2 (#51710) were obtained from the Bloomington Drosophila Stock Center (BDSC, Harvard TRiP library fly line^95^); *w*^1118^ (#60000) and *Stim* RNAi2 (#47073), *AkhR* RNAi1 (#9546) from the Vienna Drosophila Resource Center (VDRC; GD and KK library)^96^. *Stim* RNAi resistant cDNA transgenic and *Stim* cDNA RA transgenic fly lines were generated as described in supplementary information. Details on the genotypes of the fly stocks used in this study are listed in Table S1.

For density-controlled fly propagation, approximately 200 embryos were transferred into a medium food vial and 100 flies were collected 24–48 h after adult eclosion. These flies were kept in under 12:12 Light: Dark cycle and 60–70% humidity with food exchange every other day. Unless stated otherwise, all experiments were carried out on adult male flies. Details on the fly food recipes, and RNAi pulse induction regimens in adult flies are described in supplementary information.

### Quantification of body fat, glycogen, and protein levels

Unless stated differently, three to six replicates of each condition (typically five adult male flies per replicate) were collected. A coupled-colorimetric assay for TAG equivalents was carried out as previously described^97^. Glycogen level was determined with method described in Gáliková *et al*.^41^. Protein level for normalization of body fat and glycogen content was quantified with Bicinchoninic acid assay (BCA) as described in Gáliková *et al*.^58^.

### Fly body weight measurement

Three to six replicates of five adult males each were transferred into a 1.5 mL Eppendorf tube and the total weight determined using a Sartorius Microbalance MC5 (Sartorius AG, Göttingen, Germany). For dry weight, the open tubes containing flies were incubated at 65 °C for 24 hours. The empty tube weights were previously determined by averaging the values of three different measurements and subtracted from the total weight to determine the wet and dry weight of the flies. The experiment was repeated twice.

### Food intake and physiological assays

To quantify adult fly food intake, a capillary feeding (CAFE) assay was performed according to Ja *et al*.^98^ with slight modifications^31^. At least 36 flies per condition were assayed for food intake analysis. The following physiological assays were carried out based on previously described methods with minor modifications: starvation resistance assay^42^, startle-induced climbing assay^41^, and metabolic rate assay^37^; for further details see supplementary information.

### *Ex vivo* adult fly fat body tissue imaging, lipid and cell area quantification

Three to five flies per condition were analyzed. The experiment was done as described in Baumbach *et al*.^25^. Further details refer to supplementary information.

### Thin layer chromatography (TLC)

Four replicates (five adult males per replicates) were collected for TLC, which was done as described in Baumbach *et al*.^25^ with minor modifications. Experiments were repeated twice. Details are described in supplementary information.

### RT-qPCR

Three to six independent biological replicates were used. RT-SYBR qPCR was done as described in Beller *et al*.^31^. RT-Taqman qPCR followed the instructions of the kit manufacturer. Details including primer sequences on quantitative RT-qPCR are described in supplementary information.

### RNAseq analysis of adult fly fat body tissues

Abdominal fat body tissues of adult male flies at day 10 and 11 after *Stim*-TRiPI On/Off induction were dissected in cold PBS buffer, snap frozen in liquid nitrogen, and immediately stored at −80 °C. Dissected fat body tissues of about 20 flies were pooled as one biological replicate per condition of each *Stim*-TRiPI On/Off experiment. Three independent biological replicates of fat body tissues were used for total RNA isolation using a commercial RNA extraction kit (Norgen BIOTEK, Cat. #: 36200). Typical RNA samples contained at least 1 μg of RNA with concentrations around 50 ng/μL. RNA sample quality analysis, library preparation, sequencing, sequence data trimming, mapping, and reads counting were performed by the Max Planck-Genome-centre Cologne, Germany (http://mpgc.mpipz.mpg.de). Further details on RNAseq quality control, gene differential expression analysis and gene ontology analysis are described in supplementary information.

### Western blot analysis

Three independent biological replicates were used for the experiments. Primary antibodies: rabbit anti-Phospho-Akt Ser505 (1:500, NEB, Cat. #405 S), rabbit anti-Phospho-Akt Thr342 (1:500, PhosphoSolutions, Cat. #:104–342), rabbit anti-Akt (1:500, NEB, Cat. #:9272 S) and mouse anti-ß-tubulin (Developmental Studies Hybridoma Bank (DSHB); Cat. #: E7); secondary antibodies: goat anti-rabbit-HRP (1:2000, Pierce, Cat. #: 31460) and goat anti-mouse-HRP (1:10000, Pierce Cat. #31430). For further details on tissue lysis buffer and Western blot analysis see supplementary information.

### Akh and dIlp-2 immunostaining and confocal imaging

Akh and dIlp-2 immunohistochemistry was performed as described^25^ with modifications described in supplementary information.

### Circulating sugar level measurement

Circulating sugar level of adult flies was determined as described^41^ with modifications described in supplementary information.

### Statistical analysis

Unless stated differently, data were analysed by two-tailed unpaired Student’s t-tests using Microsoft Excel 2011. Statistical significance of the differences between samples is represented by asterisk: *p < 0.05, **p < 0.01, ***p < 0.001. All error bars represent standard deviations of the mean.

## Supplementary information

Xu_supplementary_information

Supplementary dataset 1

Supplementary Dataset 2

Supplementary Dataset 3
