# Supplementary material for: Chronic dysfunction of Stromal interaction molecule by pulsed RNAi induction in fat tissue impairs organismal energy homeostasis in Drosophila
Source: Sci Rep. 2019 May 6;9:6989. doi: 10.1038/s41598-019-43327-y (PMC6502815; doi:10.1038/s41598-019-43327-y)
Supplement: Supplementary file 1 — Xu_supplementary_information [file 41598_2019_43327_MOESM1_ESM.pdf]

Supplementary information

Chronic dysfunction of stromal interaction molecule by pulsed RNAi induction in fat tissue impairs organismal energy homeostasis in *Drosophila*

Yanjun Xu, Annika F. Borcherding, Christoph Heier, Gu Tian, Thomas Roeder and Ronald P. Kühnlein

Supplementary figures

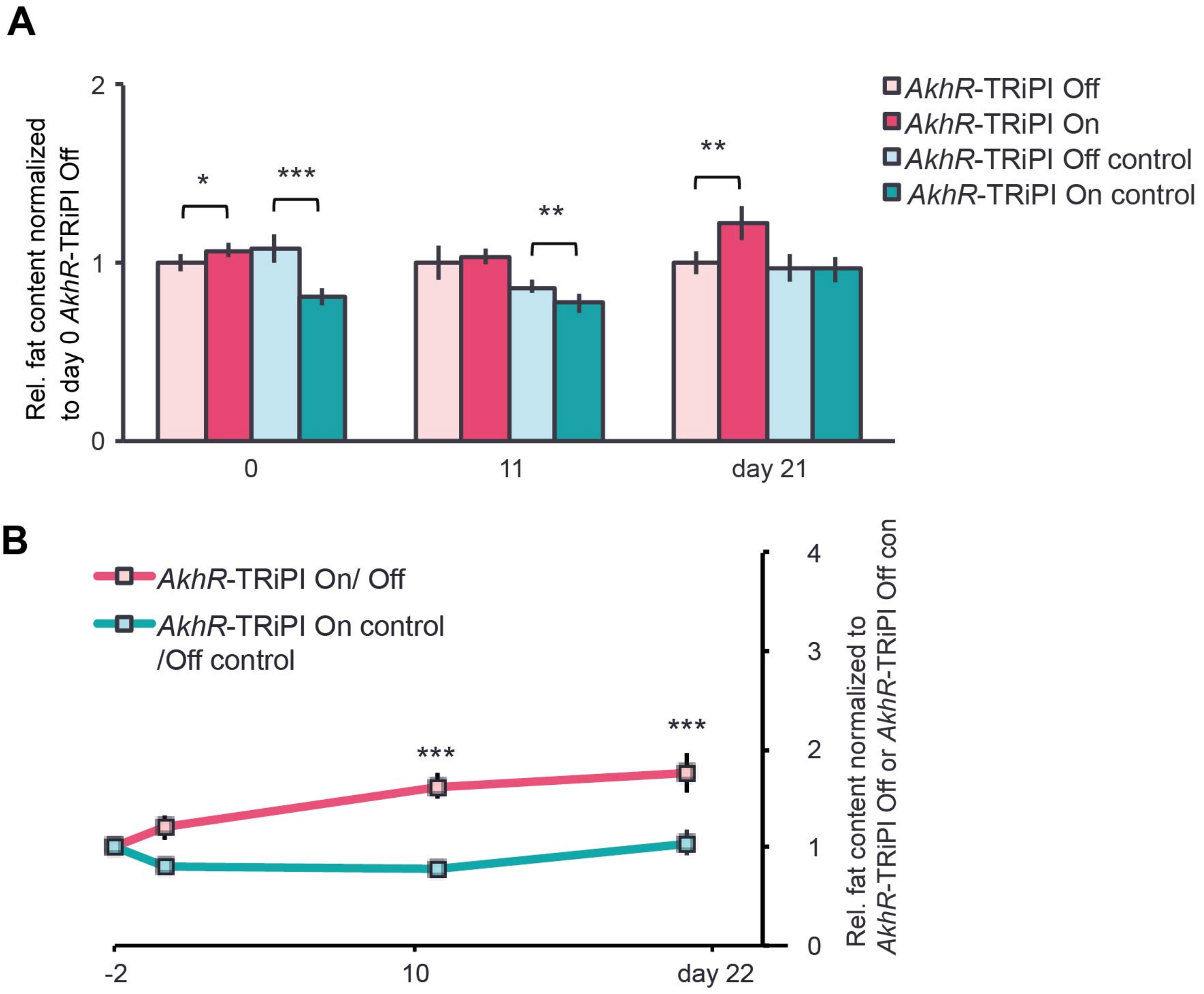

Figure S1. *AkhR*-TRiPI causes progressive body fat storage increase

(A) TRiPI with *AkhR*-short hairpin RNAi transgene (*AkhR*-shRNAi) causes minor but significant long-term increase of body fat at day 21 after *AkhR*-TRiPI On as compared to the corresponding *AkhR*-TRiPI Off flies. **(B)** *AkhR*-TRiPI On with *AkhR*- double stranded RNA

RNAi (*AkhR*-dsRNAi) causes a substantial long-term increase of body fat content at day 11 and day 21 as compared corresponding *AkhR*-TRiPI Off flies. Data are presented as means ± standard deviations from 3-6 replicates. All data were analysed by the two-tailed unpaired Student's t-test. No * $p \geq 0.05$, *$p < 0.05$, **$p < 0.01$, ***$p < 0.001$.

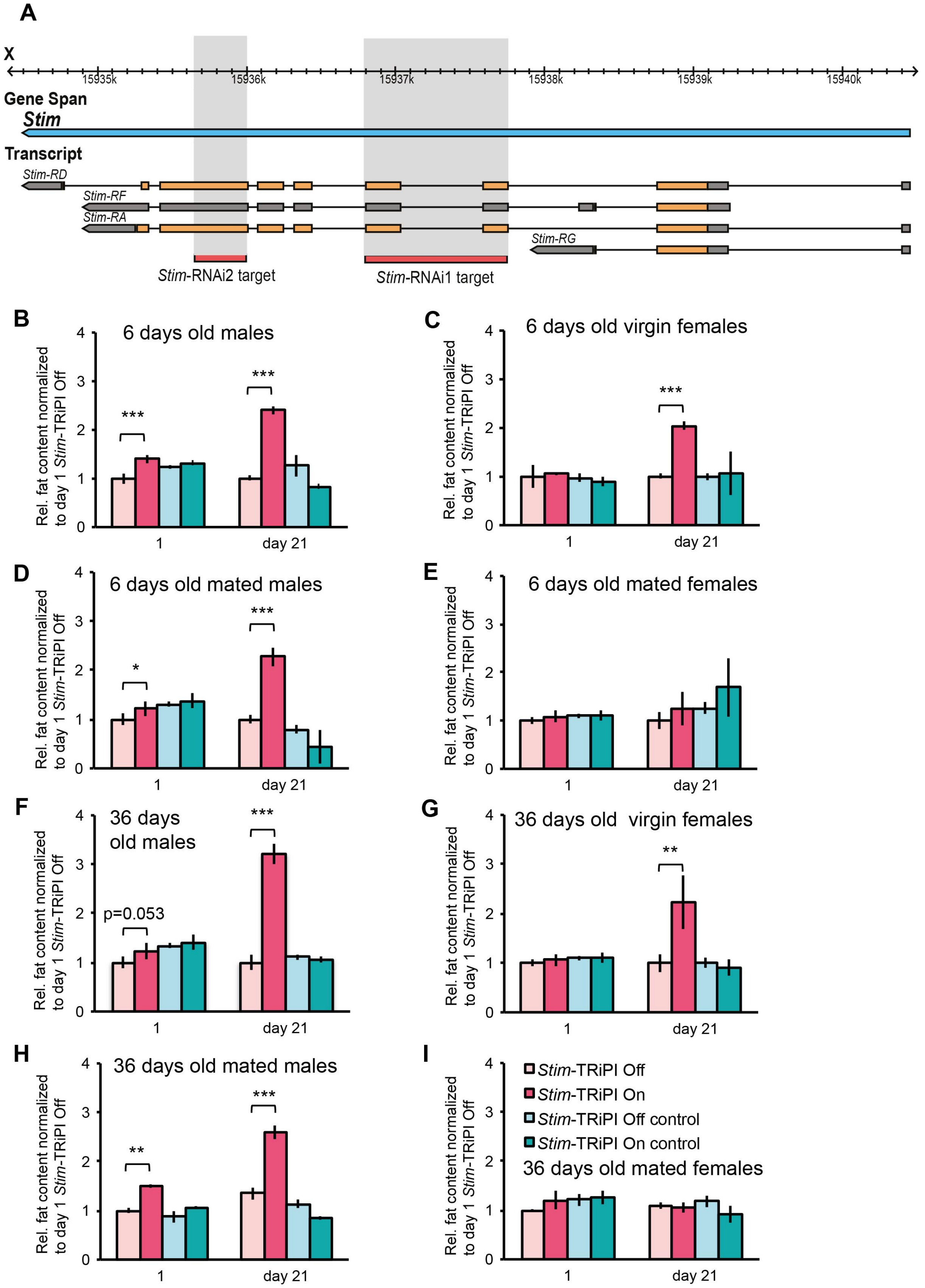

Figure S2. *Stim*-TRiPI promotes progressive body fat storage increase regardless of age and sex except for mated female flies.

(A) Gene locus scheme based on FlyBase (http://flybase.org/) showing the genomic localization of *Stim* gene (X chromosome: 15,934,400-15,940,500), the four predicted *Stim*

transcripts (*Stim*-RD, RF, RA, and RG), and the target regions of the *Stim*-RNAi1 and *Stim*-RNAi2 used in this study.

Stim-TRiPI On **(B)** in 6 days old adult male flies causes substantially increased body fat storage at day 21, kept only with males, no females present in the vials; **(C)** in 6 days old adult virgin flies causes substantially increased body fat storage at day 21, kept only with females in the vials; **(D)** in 6 days old adult male flies causes substantially increased body fat storage at day 21, flies kept together with females**;** **(E)** in 6 days old female flies causes no increased body fat storage at day 21, kept together with males; **(F)** in 36 days old male flies causes substantial increased body fat storage at day 21, no females present in the vials; **(G)** in 36 days old virgin female flies causes substantial long-term increased body fat storage, kept only with virgin females in the vials; **(H)** in 36 days old male flies causes increased body fat storage in 36 days old male flies, kept with females; **(I)** in 36 days old female flies causes no body fat storage kept with males. Relative (rel.) fat content is calculated as fold changes normalized with respect to the fat content of day 1 *Stim*-TRiPI Off control flies. Data are presented as means ± standard deviations from 3-6 replicates. All data were analysed by the two-tailed unpaired Student's t-test. No * $p \geq 0.05$, *$p < 0.05$, **$p < 0.01$, ***$p < 0.001$.

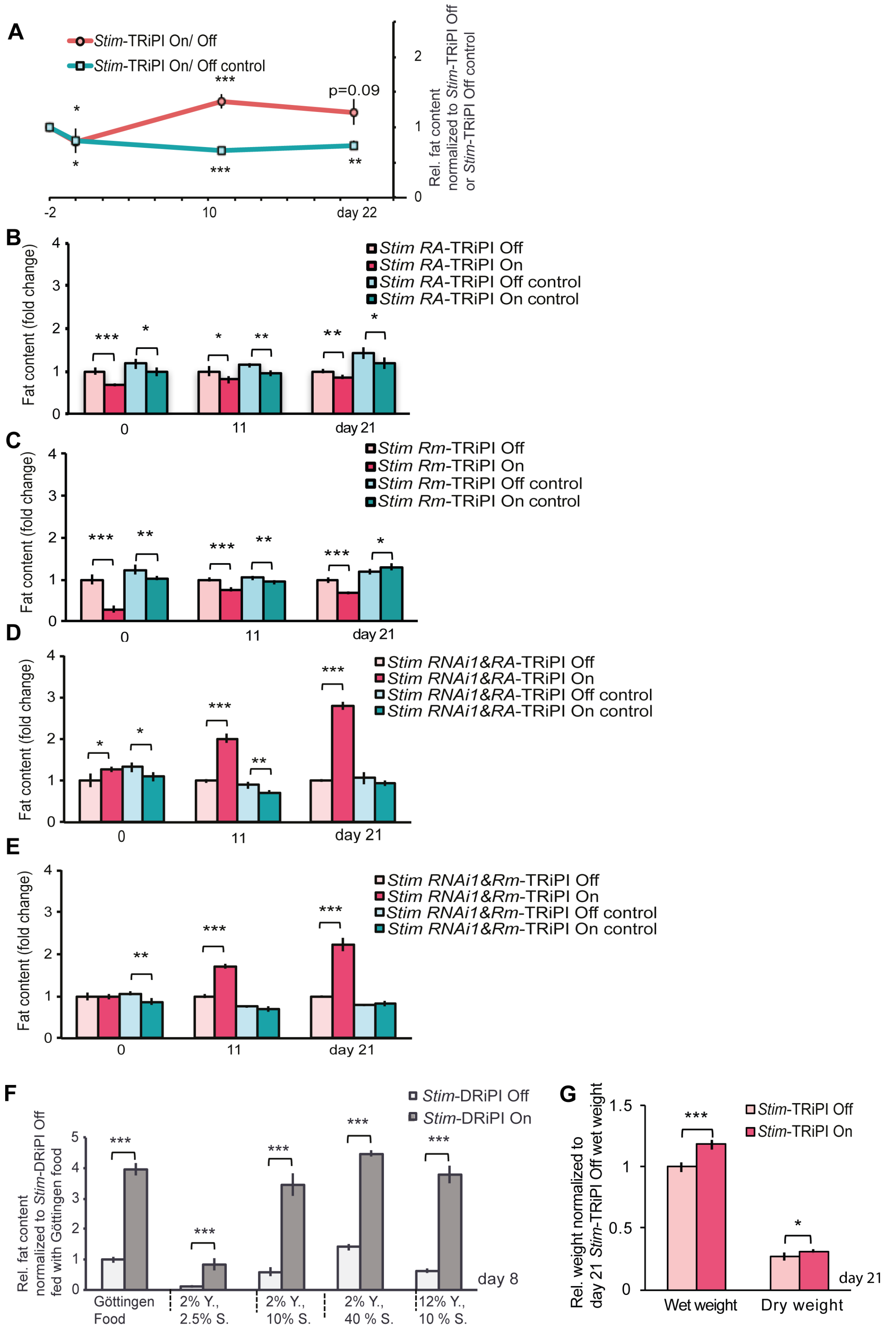

Figure S3. Specificity and diet-independence of fly obesity caused by chronic *Stim* dysfunction

(A) *Stim*-TRiPI On (using independent *Stim* RNAi2) causes long-term body fat storage increase at day 11 and day 21. Reduction of body fat content at the end of pulse induction (day 0) compared to control flies in response to *Stim*-RA (RNAi 1-sensitive *Stim* cDNA RA)-TRiPI On **(B)** and to *Stim*-Rm (RNAi 1-resistant *Stim* cDNA RA modified) **(C)**. In contrast, pulse co-induction of *Stim*-RNAi1 & RA-TRiPI On **(D)** but not *Stim* RNAi1 & Rm-TRiPI On **(E)** causes immediate body fat increase content at day 0 compared to corresponding control flies. But *Stim*-RNAi1 & RA-TRiPI On and *Stim*-RNAi1 & Rm-TRiPI On causes the long-term body fat content increase at days 11 and 21. **(F)** Diet-independent relative body fat content increase in adult male flies 8 days after *Stim*-DRiPI On. Relative (rel.) fat content is represented as fold change normalized to the value of *Stim* RNAi1 &RA TRiPI On or *Stim* RNAi1 &Rm-DRiPI On, or *Stim*-DRiPI Off flies fed with Göttingen food, respectively. **(G)** Both wet weight and dry weight of *Stim*-TRiPI adult males are increased at day 21 as compared to value of *Stim*-TRiPI Off flies. Data are presented as means ± standard deviations from 3-6 replicates. All data were analysed by the two-tailed unpaired Student's t-test. No * $p \geq 0.05$, *$p < 0.05$, **$p < 0.01$, ***$p < 0.001$.

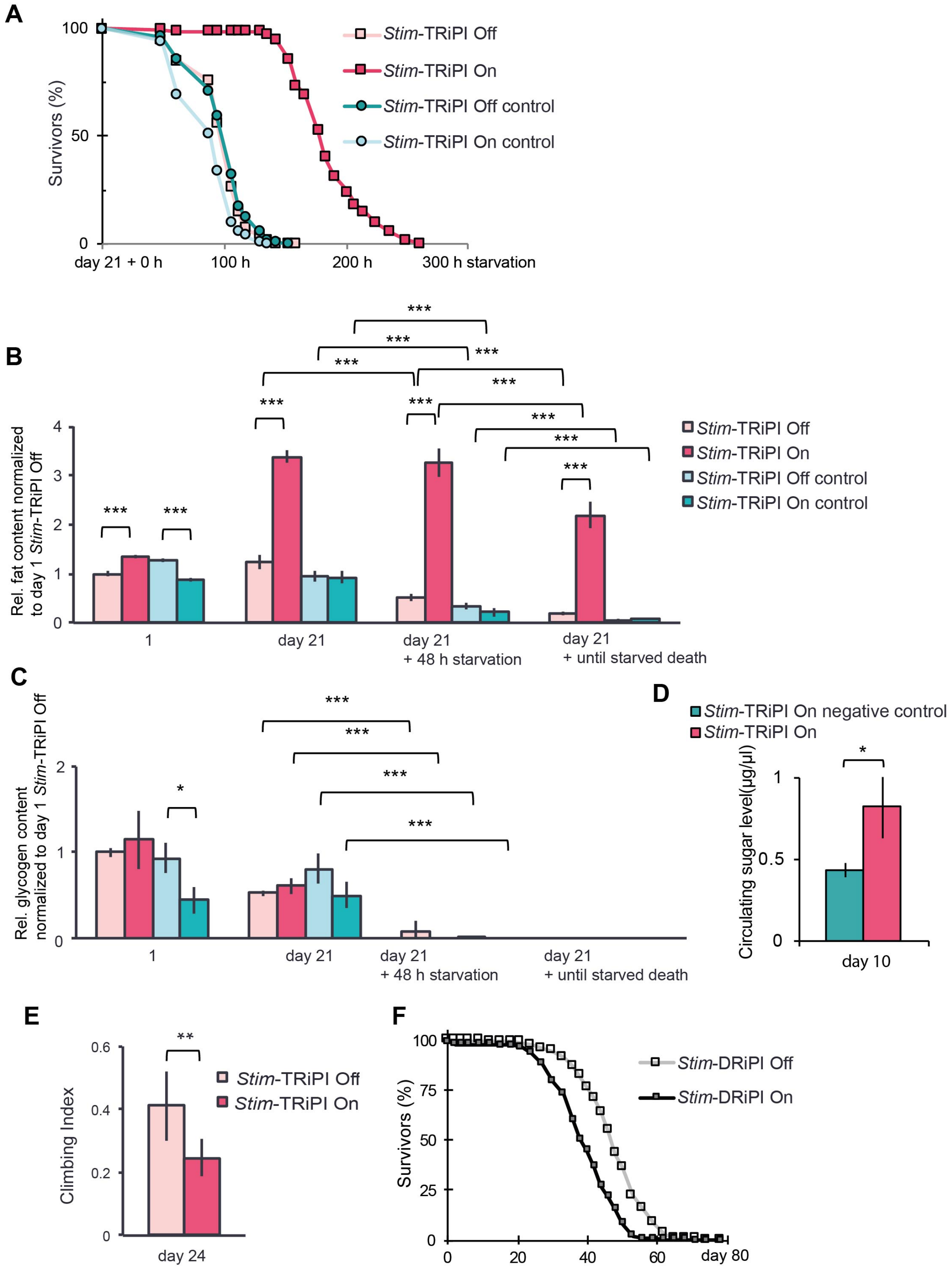

Figure S4. Obesity caused by *Stim*-RiPI affects the physiological fitness of flies

(A) *Stim*-TRiPI On significantly extends the ability of the flies to survive under starvation. *Stim*-TRiPI On / *Stim*-TRiPI Off: p<0.001; *Stim*-TRiPI On control / *Stim*-TRiPI Off control: p<0.001 (log rank test). n≥250. **(B)** Compromised lipid mobilization during short-term starvation (48

hours) and high residual *post mortem* fat content after extended starvation of *Stim*-TRiPI On adult male flies compared to controls. **(C)** Normal glycogen content and functional starvation-induced glycogen mobilization of *Stim*-TRiPI On adult male flies compared to controls. **(D)** Significantly increased circulating sugar level of *Stim*-TRiPI On adult male flies compared *Stim*-TRiPI On control (n=5). **(E)** Reduced physical fitness read out by the climbing ability of obese *Stim*-TRiPI On adult males at day 24 as compared to age-matched *Stim*-TRiPI Off flies (n=8). Data in **B-E** are presented as means ± standard deviations. All data were analysed by the two-tailed Student's t-test. No * p≥0.05, *p < 0.05, **p < 0.01, ***p< 0.001. **(E)** Reduced lifespan of obese *Stim*-DRiPI On male flies as compared to *Stim*-DRiPI Off flies (n≥200). *Stim*-DRiPI On / *Stim*-DRiPI Off: p<0.001 (log rank test).

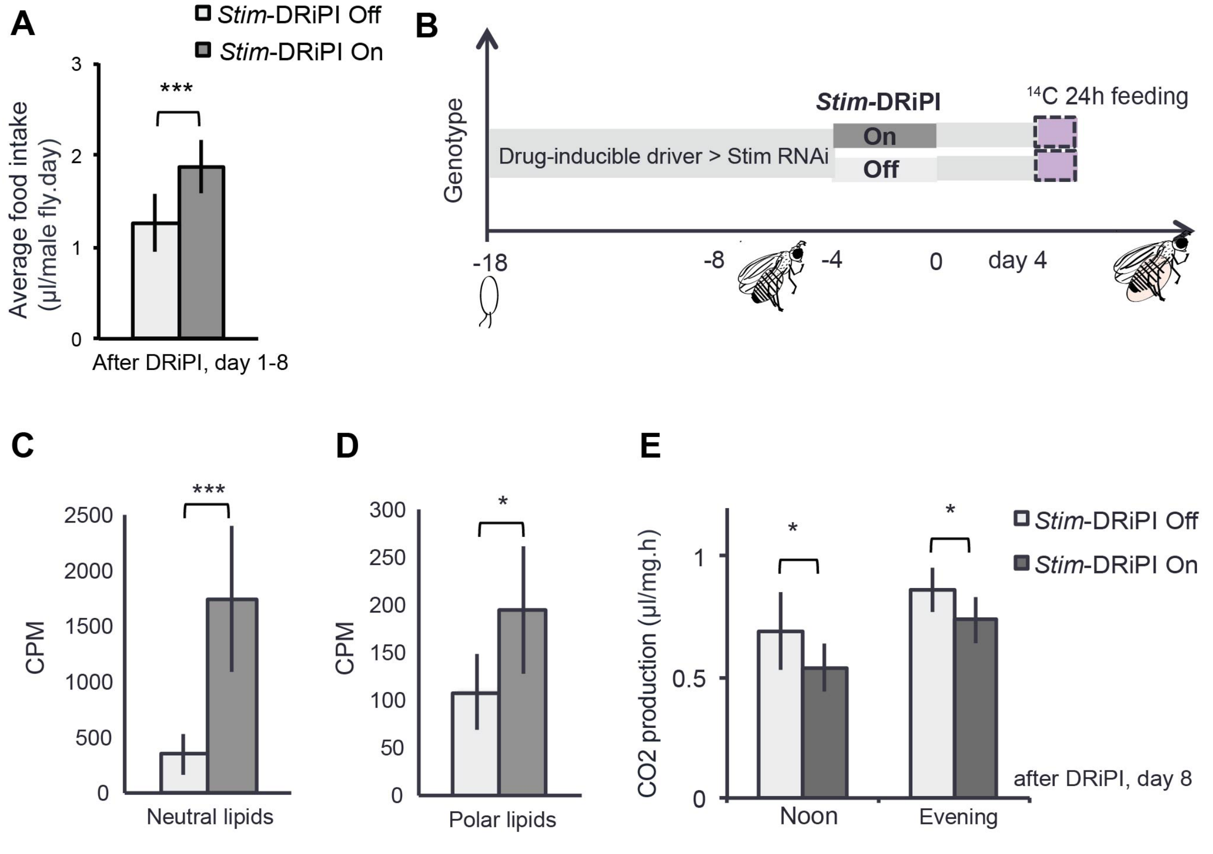

Figure S5. Hyperphagia, increased lipogenesis, and decreased energy expenditure contribute to obesity caused by chronic *Stim* dysfunction

(A) Hyperphagia of *Stim*-DRiPI On compared to the *Stim*-DRiPI Off flies illustrated by increased averaged daily food intake of males from day 1 to day 8 after pulse induction. **(B)** Experimental scheme showing the time point of pulse feeding of ^{14}C glucose labelled food for *Stim*-DRiPI On and *Stim*-DRiPI Off adult male flies. *Stim*-DRiPI On flies have a significantly higher ^{14}C incorporation into neutral lipids **(C)** and into polar lipids **(D)** than the corresponding control flies by the end of the labelling period (n=4-6). CPM = counts per minute of lipid

extractions from 20 flies. **(E)** Reduced metabolic rate of *Stim*-DRiPI On in adult males compared to *Stim*-DRiPI Off flies estimated by respirometry at noon and in the evening of day 8 after pulse induction (n=7). Data are presented as means ± standard deviations. All data were analysed by the two-tailed unpaired Student's t-test. No * p≥0.05, *p < 0.05, **p < 0.01, ***p < 0.001.

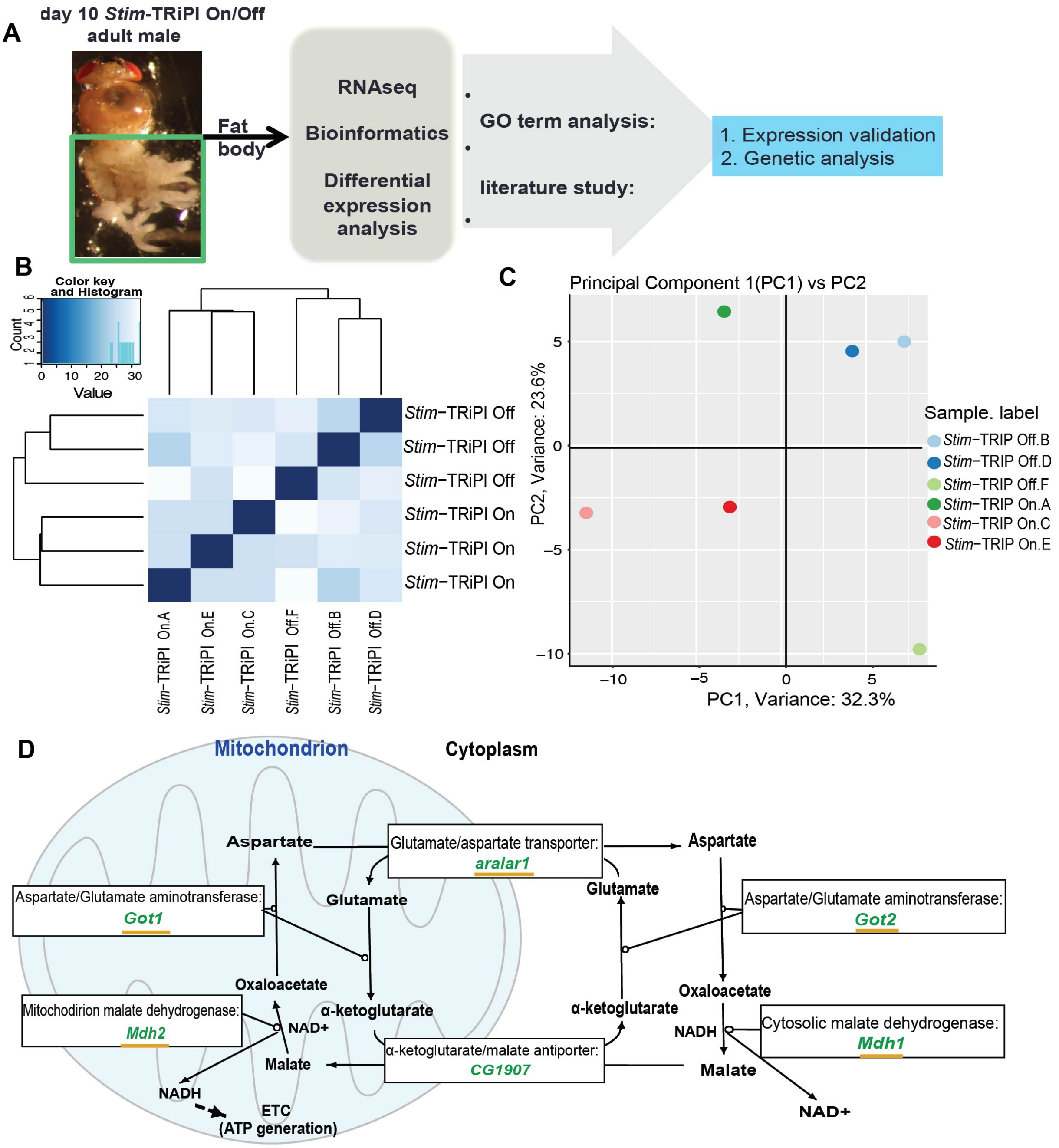

Figure S6. RNAseq analysis identifies differentially expressed *Stim*-dependent genes in fat body tissue during obesity development.

(A) Scheme showing the workflow of RNAseq analysis on abdominal fat body tissues of day 10 *Stim*-TRiPI On and *Stim*-TRiPI Off (control) male flies. Clustering **(B)** and Principal Component Analysis (PCA) **(C)** analyses confirms grouping of *Stim*-TRiPI On replicates (*Stim*-TRiPI On.A, *Stim*-TRiPI On.C, *Stim*-TRiPI On.E), and *Stim*-TRiPI Off replicates (*Stim*-TRiPI Off.B, *Stim*-TRiPI Off.D, *Stim*-TRiPI Off.F), respectively (n=3). **(D)** Schematic representation of the mitochondrial malate-aspartate shuttle. Metabolites are shown in bold black, enzymes are in boxes, green represents down-regulated genes in *Stim*-TRiPI On flies compared to *Stim*-TRiPI Off according to RNAseq. Orange underlining marks down-regulated genes which were confirmed by RT-qPCR. Data are presented as means ± standard deviations. All data were analysed by the two-tailed Student's t-test. No * $p \geq 0.05$, *$p < 0.05$, **$p < 0.01$, ***$p < 0.001$.

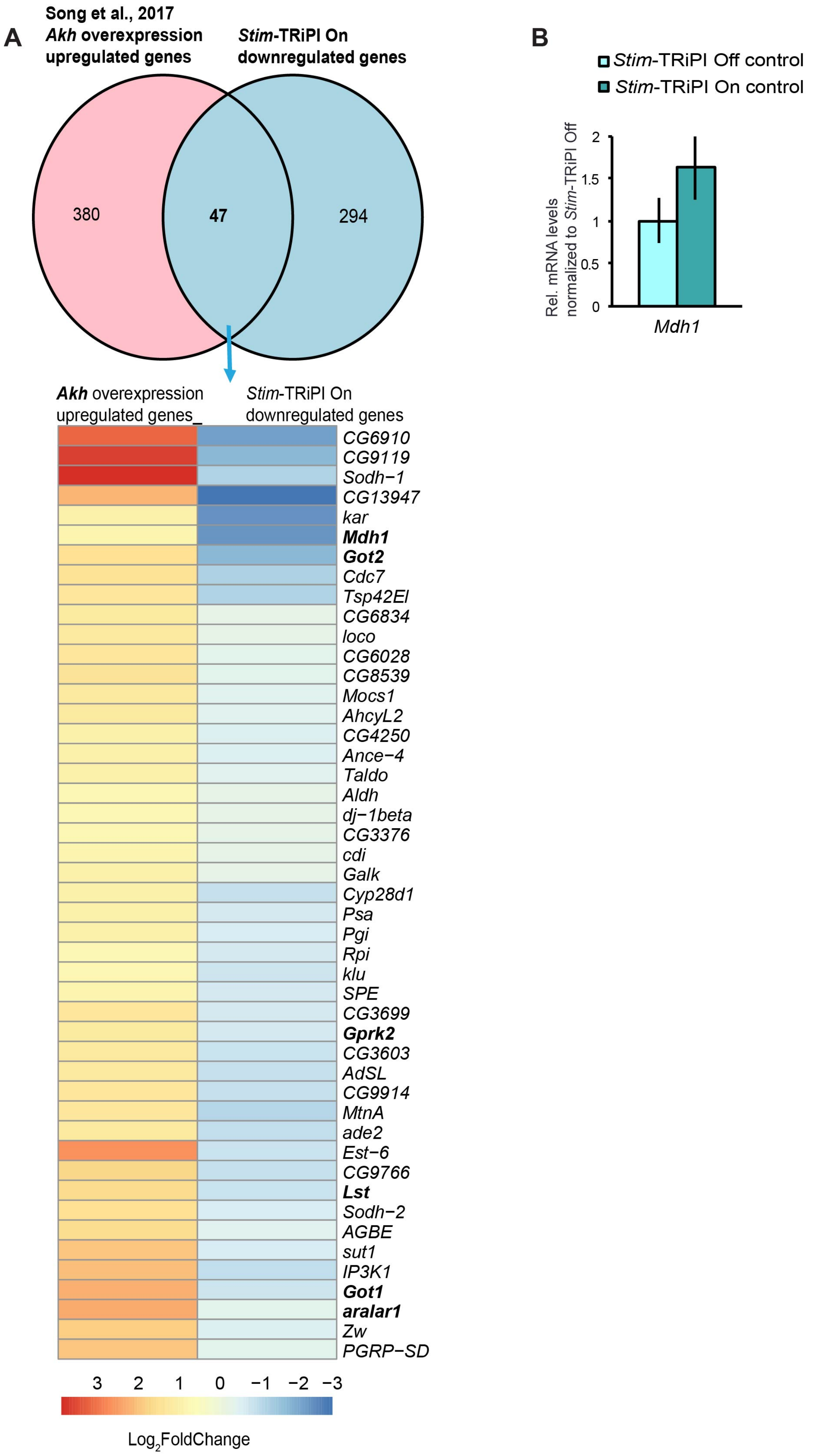
A
Song et al., 2017
Akh overexpression
upregulated genes
Stim-TRiPI On
downregulated genes
380
47
294
Akh overexpression
upregulated genes
Stim-TRiPI On
downregulated genes
CG6910
CG9119
Sodh−1
CG13947
kar
Mdh1
Got2
Cdc7
Tsp42El
CG6834
loco
CG6028
CG8539
Mocs1
AhcyL2
CG4250
Ance−4
Taldo
Aldh
dj−1beta
CG3376
cdi
Galk
Cyp28d1
Psa
Pgi
Rpi
klu
SPE
CG3699
Gprk2
CG3603
AdSL
CG9914
MtnA
ade2
Est−6
CG9766
Lst
Sodh−2
AGBE
sut1
IP3K1
Got1
aralar1
Zw
PGRP−SD
3 2 1 0 −1 −2 −3
Log2FoldChange
B
Stim-TRiPI Off control
Stim-TRiPI On control
Rel. mRNA levels normalized to Stim-TRiPI Off
0
0.5
1
1.5
2
Mdh1

Figure S7. Differential gene expression oppositly regulated by larval Akh over-expression and *Stim*-TRiPI On.

(**A**) Intersection analysis of up-regulated genes (380) by *Akh* overexpression from Song et al., 2017 RNAseq dataset and down-regulated genes (294) by *Stim*-TRiPI On identified 47 commonly regulated genes, which includes genes (*Mdh1, Got2, Got1,* and *aralar1*) involved in NADH shuttling, *Gprk2* gene involved in regulating cAMP level as well as insulin secretion suppression gene *Lst*. (**B**) Non-significant regulation of *Mdh1* in *Stim*-TRiPI On control fly fat body as compared to *Stim*-TRiPI Off control at day 1 after pulse induction (n=3-6). Data are presented as means ± standard deviations. All data were analysed by the two-tailed Student's t-test. No * $p \geq 0.05$, *$p < 0.05$, **$p < 0.01$, ***$p < 0.001$.

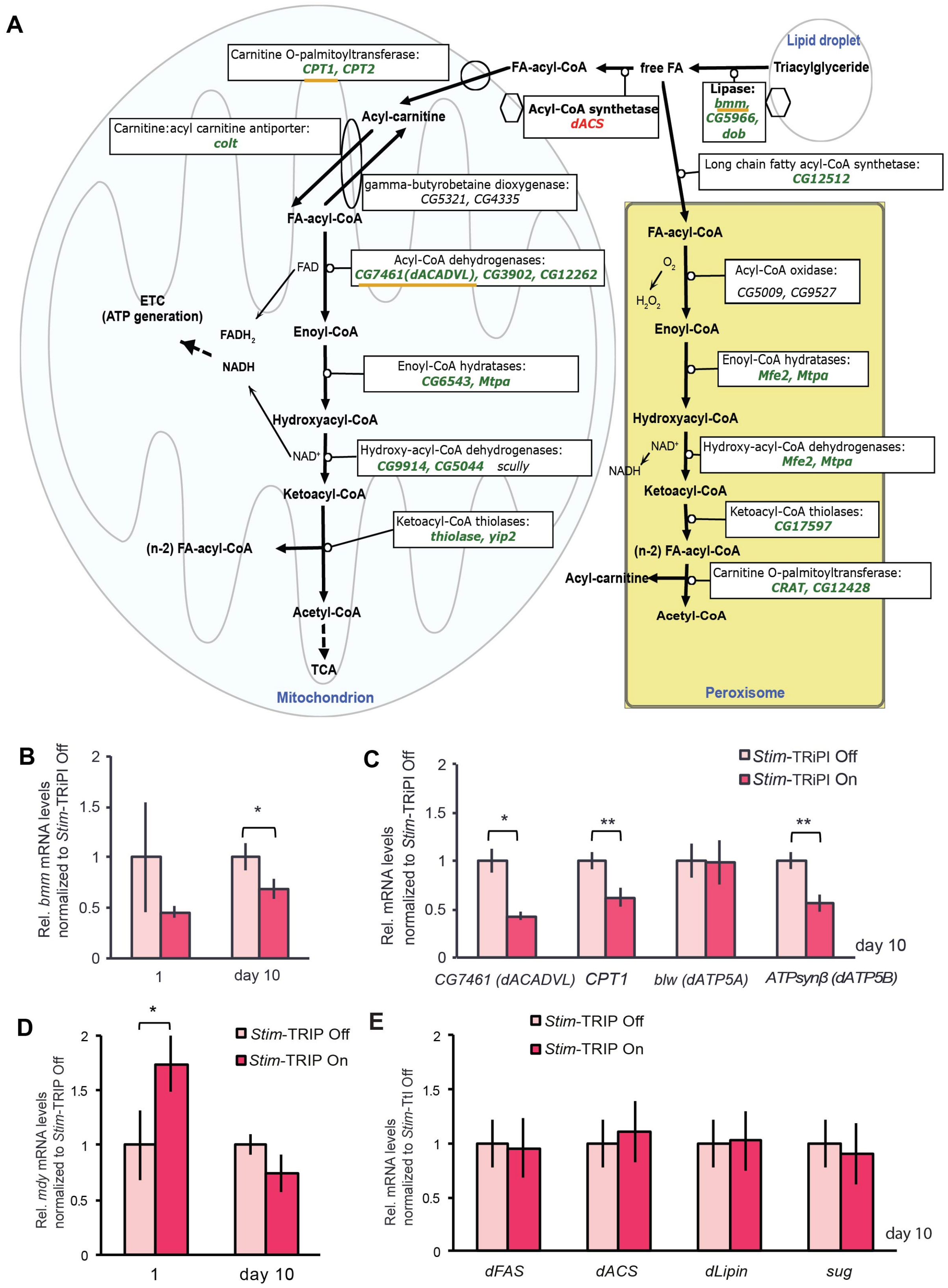

Figure S8. Repression of lipolysis and β-oxidation genes in response to *Stim*-TRiPI

(A) Schematic representation of lipolysis and fatty acid β-oxidation biochemical pathways, and their localization within the cells. Metabolites are marked in bold black, enzyme activities and the corresponding genes are in boxes. Differential gene expression according to RNAseq analysis of *Stim*-TRiPI On compared to *Stim*-TRiPI Off at day 10 are shown in red (up-

regulated), green (down-regulated), or black (unchanged). FA: fatty acid, ETC: mitochondrial electron transport chain; TCA: tricarboxylic acid cycle. The scheme was made with PathVisio. Orange underlining indicates confirmation of differential gene expression by RT-qPCR. **(B)** Down-regulation of TAG lipase gene *bmm* in *Stim*-TRiPI On fly fat body as compared to *Stim*-TRiPI Off at day 10 after pulse induction. Note that there is a trend to immediate *bmm* down-regulation already at day 0. **(C)** Down-regulation of *CG7461 (dACADVL)*, *CPT1* and *dATPsyn*β (*dATP5B*) but not of *bellwether* (*bwl; dATP5A*), in *Stim*-TRiPI On fly fat body as compared to *Stim*-TRiPI Off at day 10 after pulse induction. **(D)** Up-regulation of *mdy* , encoding an enzyme in charge of triglyceride synthesis, in *Stim*-TRiPI On fly fat body as compared to *Stim*-TRiPI Off at day 1 but not day 10 after pulse induction. **(E)** Non-significant regulation of other genes involved in lipid biosynthesis such as *Fatty acid synthase* (*dFAS), Acetyl Coenzyme A synthase (dACS), dLipin, sugarbabe (sug)* in *Stim*-TRiPI On fly fat body as compared to *Stim*-TRiPI Off at day 10 after pulse induction. **B-E**: n=3-6. Data are presented as means ± standard deviations. All data were analysed by the two-tailed Student's t-test. No * $p \geq 0.05$, *$p < 0.05$, **$p < 0.01$, ***$p < 0.001$.

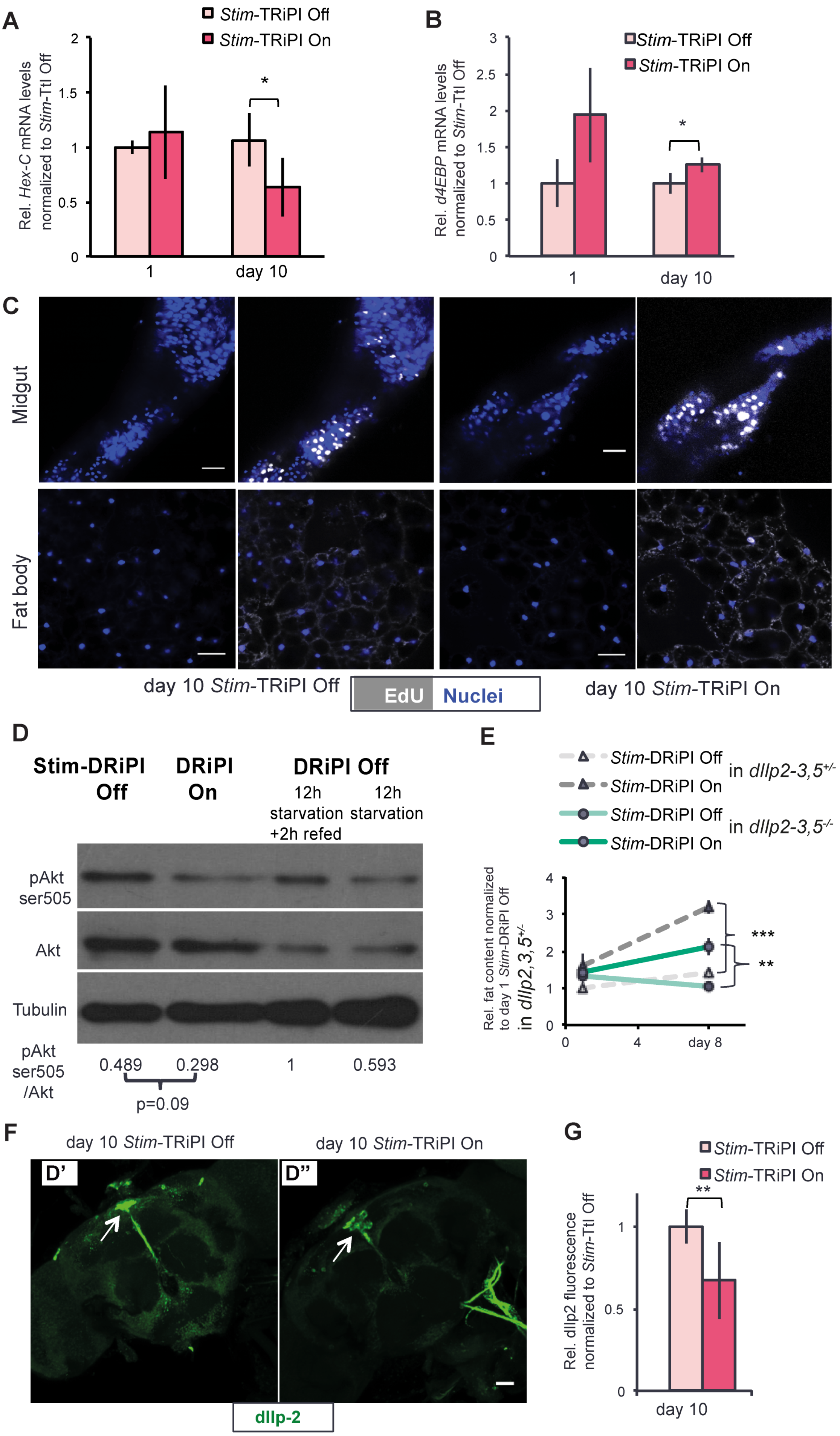
A
Stim-TRiPI Off
Stim-TRiPI On
Rel. Hex-C mRNA levels normalized to Stim-TtI Off
2
1.5
1
0.5
0
*
1
day 10
B
Stim-TRiPI Off
Stim-TRiPI On
Rel. d4EBP mRNA levels normalized to Stim-TtI Off
3
2.5
2
1.5
1
0.5
0
*
1
day 10
C
Midgut
Fat body
day 10 Stim-TRiPI Off
EdU
Nuclei
day 10 Stim-TRiPI On
D
Stim-DRiPI Off
DRiPI On
DRiPI Off
12h starvation +2h refed
12h starvation
pAkt ser505
Akt
Tubulin
pAkt ser505 /Akt
0.489
0.298
1
0.593
p=0.09
E
Stim-DRiPI Off
Stim-DRiPI On
in dIlp2-3,5+/-
Stim-DRiPI Off
Stim-DRiPI On
in dIlp2-3,5-/-
Rel. fat content normalized to day 1 Stim-DRiPI Off in dIlp2,3,5+/-
4
3
2
1
0
0
4
day 8
***
**
F
day 10 Stim-TRiPI Off
day 10 Stim-TRiPI On
D'
D''
dIlp-2
G
Stim-TRiPI Off
Stim-TRiPI On
Rel. dIlp2 fluorescence normalized to Stim-TtI Off
1
0.5
0
**
day 10

Figure S9. Insulin resistance is likely the consequence of *Stim*-TRiPI-mediated obesity. (A) Down-regulation of *Hex-C*, encoding homolog of mammalian Glucokinase in *Stim*-TRiPI On fly fat body as compared to *Stim*-TRiPI Off at day 10 but not day 1 after pulse induction. **(B)** Up-regulation of *d4EBP*, a gene suppressed by insulin signalling, in *Stim*-TRiPI On fly fat body as compared to *Stim*-TRiPI Off at day 10 but not day 1 after pulse induction **(A, B,** n=3-6**)**. **(C)** Unlike a host of EdU positive nuclei in proliferation-active tissue (midgut), EdU-positive nuclei are rare in fat body tissues of both day 10 *Stim*-TRiPI On and Off flies (n=10). **(D)** Reduced pAkt Ser505 level in *Stim*-TRiPI Off flies starved for 12h as compared to 12h starvation +2h refeeding condition indicates reduced insulin signaling (n=1). Similarly, pAkt Ser505 level show a non-significant trend to be reduced in peripheral tissues of day 4 *Stim*-TRiPI On flies as compared to *Stim*-TRiPI Off flies (n=5). **(E)** Similar as *Stim*-DRiPI On (deep gray dashed line) as compared to *Stim*-DRiPI Off (light gray dashed line) in *dIlp2-3,5* heterozygous flies, fat content level significantly increased in the following 8 days after *Stim*-DRiPI On (deep green) as compared to *Stim*-DRiPI Off (light green) in *dIlp2-3,5* homozygous mutant flies (n=6). Relative (rel.) fat content is represented as fold change normalized with respect to the value of day 0 *Stim* TRiPI Off in *dIlp2,3,5* heterozygous flies (n=6). **(F)** Immunostaining of dILP2 peptide with anti-dILP2 antibody (Green) in insulin producing cells (IPCs, indicated by white arrow) of *Stim*-TRiPI On and Off fly brains at day 10. **C, F:** Scale bar = 25μm. **(G)** dILP2 staining intensity is decreased in IPCs of *Stim*-TRiPI On flies as compared to *Stim*-TRiPI Off flies at day 10 (n=8-10). Data are presented as means ± standard deviations. All data were analysed by the two-tailed Student's t-test. No * $p \geq 0.05$, *$p < 0.05$, **$p < 0.01$, ***$p < 0.001$.

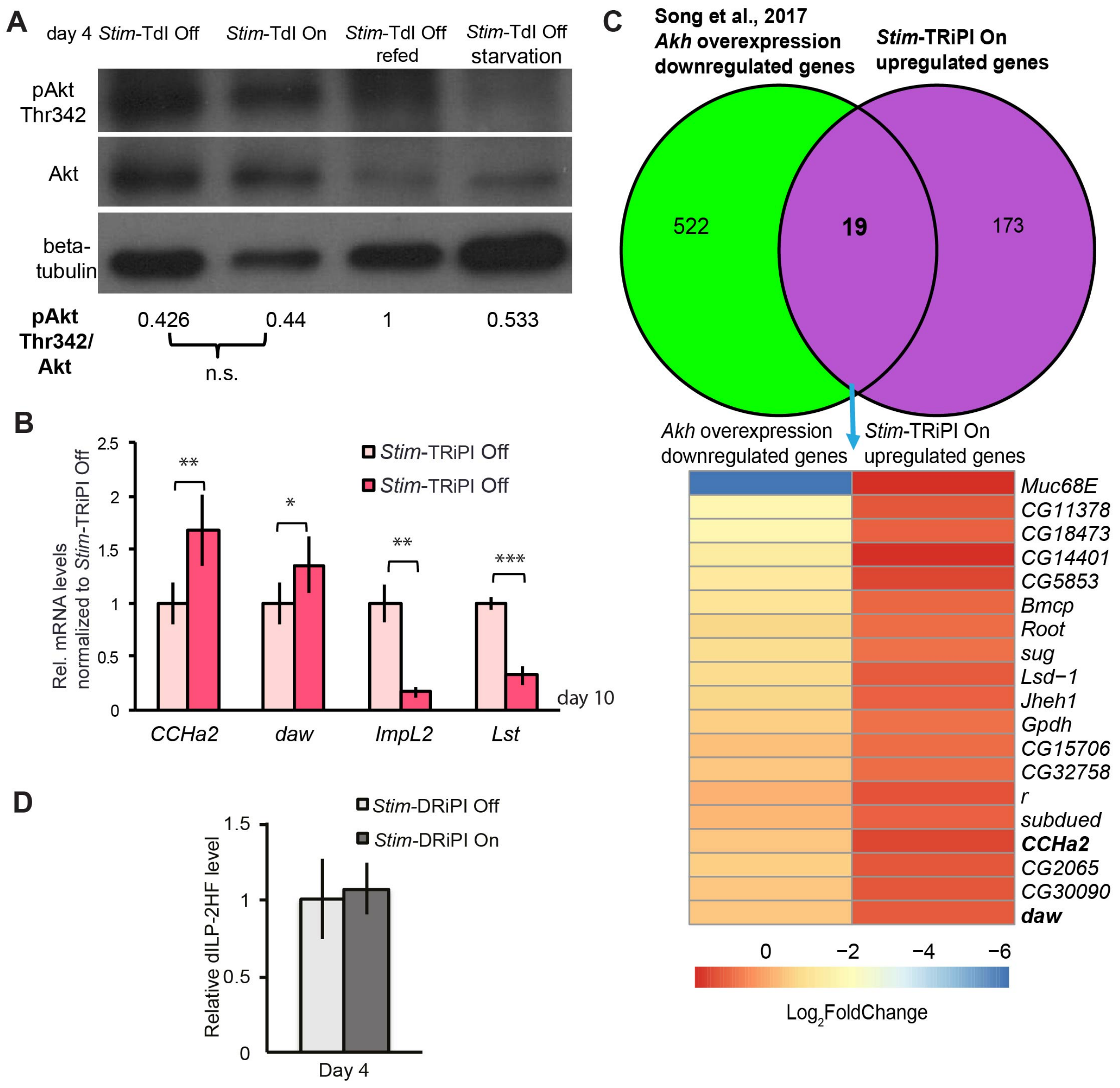

Figure S10. pAkt Thr342 level, intersection in obese Stim-DRiPI flies and intersection of the expression analysis comparing down-regulated genes in resonse to *Stim*-TRiPI to up-regulated genes in response to larval *Akh* overexpression.

(A) Unlike reduced pAkt Thr342 level in *Stim*-TRiPI Off flies starved for 12h as compared to 12h starvation +2h refeeding condition (n=1), pAkt Thr342 level was not changed in peripheral tissues of day 4 *Stim*-TRiPI On flies as compared to *Stim*-TRiPI Off flies (n=5). **(B)** Differentially expressed genes encoding candidate secreted proteins: up-regulation of *CCHa2* and *daw*, but down-regulation of *ImpL2* and *Lst* in in *Stim*-TRiPI On fly fat body as compared to *Stim*-TRiPI Off at day 10 after pulse induction (n=3-6). **(C)** Intersection analysis of down-regulated genes (522) in response to *Akh* overexpression (from Song et al., 2017 RNAseq dataset) and up-regulated genes (173) in response to *Stim*-TRiPI On identifies 19 commonly regulated genes, which include genes encoding secreted factors (*CCHa2, daw*). **(D)**. Circulating dIlp2HF level is unchanged in day 4 *Stim*-DRiPI On flies as compared to the value of *Stim*-DRiPI Off flies

(n=8). Data are presented as means ± standard deviations. All data were analysed by the two-tailed Student's t-test. No * p≥0.05, *p < 0.05, **p < 0.01, ***p < 0.001.

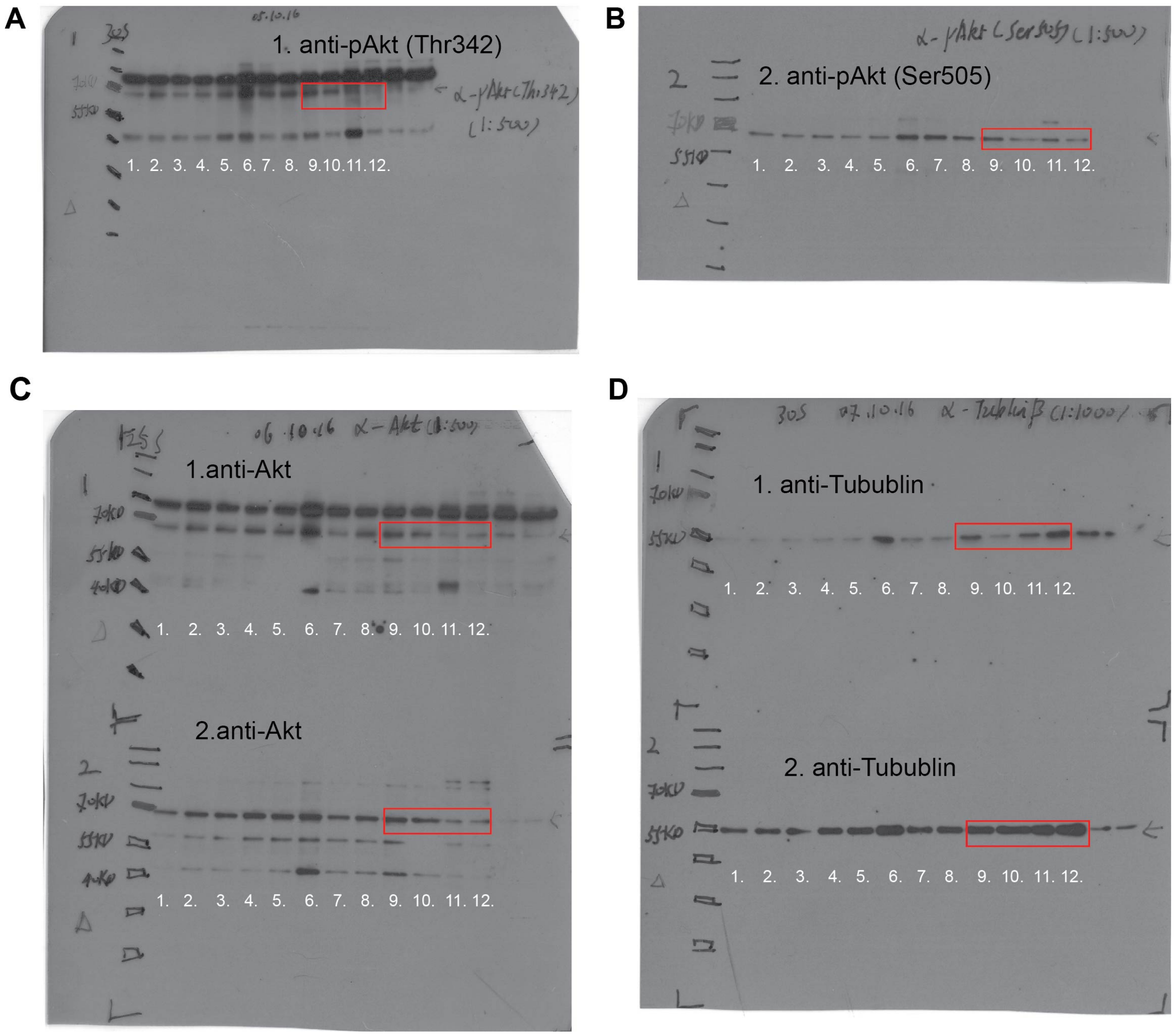

Figure S11. Whole gel image of Western blot on *Stim*-DRiPI On and Off abdomen protein samples.

Red square indicates the representative cropped image shown in **figure S9D, S10A** in whole gel image of western blot targeting on phosphorylated Akt at Thr342 and Ser505– around 70KDa protein band (**A, B**), 70KDa total Akt band (**C**), and the corresponding loading control protein 55KDa beta-tubulin (**D**), respectively. Sample information are listed in **table S4.**

Supplementary materials and methods

Fly food recipes

Except stated differently, flies were raised on standard "Göttingen food" (Gö-food; 20 L H2O, 125 g agar, 360 g dry yeast, 200 g soy flour, 440 g treacle, 1.6 kg cornmeal, 1.6 kg malt, 125

mL propionic acid and 30 g nipagine) within midsize vials ("food vials"; Greiner Bio-One, Cat. #217101) into which a filter paper and a mite sponge plug were inserted sequentially. Alternatively, in feeding experiments with different dietary composition, flies were kept on food containing a mixture of yeast (=Y.; 2% or 12% weight/weight (w/w)) and Sucrose (= S.; 2.5% to 40% w/w) in 1% agar in water. For embryo seeding, virgin female and males were kept in a cage with apple juice agar plate (30% apple juice volume in 1% agar and water solution) plus yeast for overnight embryo collections.

RNAi Pulse Induction in adult flies

For **R**NAi **P**ulse **I**nduction (**RiPI**) in adult flies, virgin female flies containing the tissue-specific, temperature-sensitive Gal4 fat body driver (ts-FB-Gal4, based on TARGET system[1]) were mated with males of the indicated UAS-RNAi transgenic lines. In parallel, control (*w*^{1118}) virgins were crossed with the same UAS-RNAi transgenic lines. Offspring flies were kept at 18°C to repress RNAi activation. On day six after eclosion, the flies encoding both the RNAi and the Gal4 driver transgenes were exposed to 29°C (34-48 hours) to induce **T**emperature-sensitive **R**NAi **P**ulse **I**nduction (**TRiPI On**). Flies of identical genotypes, which were kept at 18°C, are referred to as **TRiPI Off**. Additional control flies, which encoded the UAS-RNAi transgene construct but no Gal4 driver were also exposed to 29°C for 38 hours referred to as **TRiPI On control**; Genotype-identical flies, which were not subjected to the temperature shift, are referred to as **TRiPI Off control**.

For **D**rug-sensitive **R**NAi **P**ulse **I**nduction (**DRiPI**), virgin females containing the drug-inducible ubiquitous driver daGS[2] were crossed to male flies encoding an UAS-RNAi. After offspring eclosion, four days old adult male flies encoding both transgenes were fed either with the drug-supplemented food (Gö-food containing 200µM mifepristone (RU-486); Sigma-Aldrich, Cat. #: M8046-500MG, pre-dissolved in ethanol; **DRiPI On)**, or control food (Gö-food supplemented with the equivalent volume of ethanol; **DRiPI Off**) for four days. After drug/control food feeding, flies were kept on the regular Gö-food for time periods reported in the results section.

Table S1 Fly stocks

Short Name	Stock Number	Genotype	Reference/ Source
w-control	RKF1084	*w[1118]; +/+; +/+*	VDRC60000
ts-FB-Gal4	RKF805	*y* w*; P{w[+mW.hs]=GawB}FB P{w[+m*] UAS-GFP 1010T2}#2; P{w[+mC]=tubP-GAL80[ts]}2*	[3]

daGS	MGF1663	*w[1118]; daGS; +/+*	[2]
Stim RNAi1	RKF1112	*w[1118]; UAS dSTIM[RNAi] / CyO float; +/+*	[4]
Stim RNAi2	RKF1178	*w[1118]; +/+; P{GD16187}v47073*	VDRC47073
AkhR dsRNAi	YXF1410	*w[1118]; +/+; P{GD586}v9546/TM3 Sb*	VDRC9546
AkhR shRNAi		*y[1] v[1]; P{y[+t7.7] v[+t1.8]=TRiP.HMC03228}attP40; +/+*	BDSC51710
Akh^A	MGF1639	*w[1118]; akhp-GAL4, UAS-mCD8 GFP /CyO; Akh[A]/ TM3 Ser**	[5]
daGS; Akh^A	YXF1765	*w*; daGS/CyO; Akh^A/TM3 Ser**	This study
Stim RNAi1; Akh^A	YXF1766	*w*; StimRNAi1/CyO; Akh^A/TM3 Ser**	This study
balancer line 1	RKF891	*w*; wg[Sp-1] / CyO P{ry ftz-lacZ} ; D* / TM3 Sb*, P{w[+] Ubx-lacZ}*	Alf Herzig
balancer line 2	RKF1365	*w[*]; Kr[If-1]/CyO; D[1]/TM3, Ser[1]*	BDSC7198
Stim-Rm	YXF1569	*y[1] M{vas-int.Dm}ZH-2A w*; +/+; RNAi1-resistant Stim-Rm/ TM3 Sb**	This study
Stim-RA	YXF1574	*y[1] M{vas-int.Dm}ZH-2A w*; +/+; RNAi1 sensitive Stim-RA/ TM3 Sb**	This study
Stim RNAi1; Stim-Rm	YXF1870	*w*; StimRNAi1/ CyO; RNAi1- resistant Stim-RA modified/ TM3 Ser**	This study
Stim RNAi1; Stim-RA (Stim RNAi1&RA)	YXF1868	*w*; StimRNAi1/ CyO; RNAi1 sensitive Stim-RA/ TM3 Ser**	This study
dIlp2-3,5 mutant	RKF1661	*w*; +/+; dIlp2-3,5/TM3 Ser*,* PP{w[+]Act-GFP} float.	[6]
daGS; dIlp2-3,5 mutant	YXF1757	*w*; daGS/CyO float; dIlp2-3,5 mutant/TM3Ser**	This study
Stim RNAi1; dIlp2-3,5 mutant	YXF1758	*w*; StimRNAi 1/CyO float; dIlp2-3,5 mutant/TM3Ser**	This study
daGS; $Ilp2^1$ gd2HF		*w*; daGS/CyO float; daGS; $Ilp2^1$ gd2HF /TM3Ser**	This study
Stim RNAi1; $Ilp2^1$ gd2HF		*w*; StimRNAi 1/CyO float; $Ilp2^1$ gd2HF /TM3Ser**	This study

Generation of transgenic fly lines

The strategy and procedure designed for the molecular cloning of the *Stim*-RNAi1-resistant cDNA construct, was kindly provided by Dr. J. Baumbach. First, the *Stim*-RNAi1 targeted sequence (344bp, in green capital letters) was altered into a RNAi-resistant sequence by replacing the nucleotides in the third position of each amino acid code into a different

nucleotide that codes for the same amino acid that is on the basis of the *Drosophila* codon usage (see http://www.kazusa.or.jp/codon/cgibin/showcodon.cgi?species=7227). Together with the adjacent sequence (in bold black letter) and convenient endonuclease restriction sites at the 5' (KpnI) and the 3' end (AhdI) (shown in red capital letters), respectively, a *Stim*-RNAi1 resistant *Stim*-cDNA RA modified fragment (812bp, refer to sequence shown below) was custom-synthesized and cloned into cloning vector pEX-A (Eurofins MWG Operon). The *Stim*-RA cDNA generated by the Berkeley *Drosophila* Genome Project (BDGP) [7] was obtained as a pOT*Stim*-RA clone (LD45776) from the *Drosophila* Genomics Resource Center (DGRC). The *Stim* wild type sequence of the *Stim*-RA clone was replaced with the *Stim*-RNAi1 resistant *Stim*-cDNA RA modified fragment (cut at the 5' KpnI and 3'AhdI sites) to generate the *Stim* RNAi1 resistant cDNA RA modified donor vector called pOT *Stim*-Rm. Finally, both the pOT *Stim*-RA cDNA and pOT *Stim*-Rm were cut by BglII and XhoI and the inserts cloned into the corresponding restriction sites of the pUASTattB vector [8]. The resulting transgene constructs were injected by BestGene Inc. into *y[1] M{vas-int.Dm}ZH-2A w[*]; M{3xP3-RFP.attP}ZH-86Fb* (BDSC24749) embryos for site-specific integration at cytogenetic position 86F8 on the 3rd chromosome. For details on clones see table S2.

Sequence of the synthetic *Stim*-RNAi1-resistant *Stim*-RA cDNA called *Stim*-Rm

5'gaaactGGTACC**cgctggaaagactacatattggtaacactgttgcttagtgctattattggttgttggtacgcctatc agcaaaataagaatgccaaacggcatctgcgtcgaatggcccaggatatggagggattgcagagggctgagcaaag tctacaggagatgcagaaggaactagaacgg**GCAAGGATGGAACAAGAGAACGTAGCTACTAGAA GCTTGACTTAGAACGACGACTTAAGGAGGCACCGACACTGAGCTCTTCCAATTCCGACT TAGAGGTCCAACAACTCAAGAAAGAGATTGAAATGTTACGGAATGAGTTATCGCGGGCG GAGTTTGAACTGGTTGATAATTGTTGGTCTCCACCCCCGCAGCTCCAGTCTTGGCTACA GTATACCTACGAGCTTGAGAGAAAAAACCACCAAAAGAAACGGACCTCCGCGGAAAAAC AACTCCAATCCGCGAGGGAAGCGTGCGAAAAGTTACGCAAAAAGCGCTCTAGCTTAGT CGGAGCCTTTGTCTCG**acgcacggaaagagtattgatgatgtggatcggtcgattgttgaggcacggaatgcc ctcggagatgtaacaaacgagctgcaagaacgactgcatcgctggaagcaaatcgagacgtgccttggcttaaacatt gtgaacaacaatggtctgccctacttggagaatgttctgtacggtcgaaatgggggcttacaaagttccatgggcatga gttcaaccaagggttctagagcacgtattaccaacagcaccgaagacctg**GACGATGAGTCcataca 3'

Table S2 Plasmids used in this study

**Internal stock number**	**Construct**	**Vector Backbone**	**Reference/ Source**
YX460a	pEX-A *Stim-Rm*	pEX-A (2450 bp)	Eurofins MWG Operon
YX461a	pOT2 *Stim-RA*	pOT2 (1665bp)	[7]

			BDGP: LD45776
YX462	pOT2 *Stim-Rm*	pOT2 (1665bp)	This study
RK416	pUASTattB *LSD-1-PC:EGFP*	pUASTattB (8489bp)	[3]
YX463	pUASTattB *Stim-Rm*	pUASTattB (8489bp)	This study
YX464	pUASTattB *Stim-RA*	pUASTattB (8489bp)	This study

Ex vivo adult fly fat tissue imaging and lipid staining

After quick CO_2 anaesthesia, wings and legs of adult males were removed. Fly bodies were further kept on ice for 10 min. The fly bodies were put on a metal plate together with a plastic ruler, which were magnified by Zeiss Stereomicroscope Discovery V8 microscopy (Carl Zeiss AG, Jena, Germany) and imaged by a Canon EOS 5D Mark II camera (Canon, Tokyo, Japan).

For abdominal fat body tissue imaging and dissection, flies without wings and legs were further fixed on a silica bed with insect pins and dissected in cold PBS. Then fly fat body tissues were directly mounted in PBS and directly imaged or stained in PBS containing LD540 (1:500, gift from C. Thiele[9]) for lipid droplet staining, CellMask (1:1000, Thermo Fisher Scientific, Cat. #C10046) for cell membrane staining, and DAPI (1:1000, Thermo Fisher Scientific, Cat. #D1306) for nuclear staining. After 5 min incubation, the fat tissue was washed with PBS once, and mounted using round cover slips (diameter=10mm, Thermo Fisher Scientific, Cat. #631-1340). The brinks of cover slips were sealed by nail polish. After that, confocal imaging was carried out with Zeiss Laser Scanning Microscope (LSM) 780 confocal microscope system (C-Apochromat 40x/1.20 W Korr. M27 objective, fixed gain, 8 bits, pixel size: 2048 x 2048, 1 optical stack in Z-axis direction). Fat body tissue of at least 3 flies per condition or genotype were dissected and analysed by Fiji (image J, 1.51h) [10,11]. Experiments were repeated twice.

Fat body cell and lipid droplet size quantification

Intact fat body cells, which have the DAPI signal marking their nuclei but no apparent lipid droplet fusions, were selected. Cell area sizes based on CellMask signals were quantified with the polygon selection tool, and the LD540 signal channel image of selected cells were duplicated and saved for lipid droplet size quantification. To exclude disturbing signals of cells, images (cells of interest, LDs image channel) were first copied into a new image with black background (1,000x1,000 pixels for normal cell size). After that, the black background images were processed by “Gaussian blur” with sigma (radius) 2.00 for more smooth signals. Subsequently, the smoothed images were processed with “Enhance Local Contrast” (Contrast Limited Adaptive Histogram Equalization, CLAHE) for better local contrast (block size: 127,

histogram bins: 256, maximum slope: 3.00, non-mask, normal process). Then, the minimum displayed values of images were adjusted to optimized threshold. Low intensity signals were increased, while high intensity signals were not enhanced to improve signal-to-noise ratio and signal homogeneity of candidate lipid droplets (strongly and weakly stained). Moreover, the images were processed with “Gaussian blur” again. Later, binary images were generated based on above step processed images (Method: Moments, Visualization: Black and white). The candidate lipid droplet areas were separated by “watershed” and verified by manual separation (Inverted picture, separate by black pencil, 2 pixels, 71 width). Finally, binary images with lipid droplet objects were analysed by “analyze particles” (size: 0.1 µm -infinity, circularity: 0.01-1, show: masks, selecting choices: Display results, clear results, Summarize, Add to Manager). This way, averaged cell area, total lipid droplet area per cell, averaged lipid droplet size per cell in each fly fat body tissue were obtained. Moreover, averaged lipid droplet area percentage of each cell in fly fat body tissues was calculated. Data were statistically analysed with Excel 2011 (Student’s t-test).

Thin layer chromatography (TLC) and *de novo* lipogenesis analysis

Typically, lipids of one fly were loaded for each TLC replicate. TLC experiments were done as described in Baumbach et al., 2014 [12]. Density-based TAG quantification was done with Fiji (ImageJ, 1.51h).

For lipogenesis analysis, two groups of day 4 *Stim*-DRiPI On and Off male flies were transferred to Gö-food, which contained ^{14}C labelled glucose (Perkin Elmer, Cat. #NEC042X050UC), dissolved in ethanol; each 1mL Gö-food mixed with 18.3 µL ^{14}C glucose solution. 100 flies were kept in a medium-size vial with about 200µl food. After 24 hs feeding, at least 80 flies were frozen in liquid nitrogen (at least 4 replicates of 20 flies each per replicate) using a 1.5 mL Eppendorf tube. Flies were homogenized in 0.5 mL chloroform with pestle using a motor-driven homogenizer (VWR, Cat. #: 431-0094 and 431-0100). Then lipid fractionation was performed following the solid phase extraction method described by Kaluzny et al.1985 [13]. Fly homogenates were transferred onto chloroform pre-equilibrated DSC-NH2 columns (PK30 DISCOVERY DSC-NH2 6mL Tube 1GM, Sigma-Aldrich, Cat. #52640-U). The following lipid fractions were eluted sequentially with 3 mL chloroform: 2-propanol (2:1) solvent to elute the neutral lipids (TAGs, DAGs, cholesterol). After washing the column with 3 mL diethyl ether containing 32% acetic acid, 3 mL methanol was used to elute the phospholipids and fatty acids. The solvents were evaporated under nitrogen. The dried neutral lipids as well as the combined phospholipid and fatty acid fractions were dissolved in 4 mL scintillation liquid (Ultra Gold scintillation cocktail, Perkin Elmer, Waltham, USA, Cat. #6013326) and scintillation samples were transferred into 4 mL plastic scintillation vials. Radioactivity (counts per minute, CPM)

was measured in a scintillation counter (Tri-Carb 2100TR, Packard, Perkin Elmer; each sample for 4min counting). Unless stated differently, other related chemical reagents were from Merck. The experiment was repeated twice.

RT-qPCR

For SYBR Green qPCR (Rotor-Gene SYBR Green PCR Kit, Qiagen, Cat. #204076), ten adult male flies per biological replicate (at least 3 biological replicates) were collected for whole fly head or abdomen RNA extraction. Fat body total RNA sample were isolated from abdominal fat body tissues of 12-15 adult male flies (6 biological replicates). RNA extractions were done on with Quick RNA MicroPrep Kit (Zymo Research, Cat, #R1054). Identical amounts of RNA (whole fly or head: 1 µg; fat body tissue: 500 ng) were used for reverse transcription using the QuantiTect Reverse Transcription Kit (Qiagen, Cat. #205311).

Act5C and/or *Ribosomal protein L32* (*RpL32*) gene expression were used for normalization [14,15]. qPCRs were performed with three technical replicates of each cDNA sample using standard running protocols: 95 °C 5 min plus 40 amplification cycles (95°C and 60°C 10 s) on a QIAGEN Rotor-Gene Q system or Applied Biosystems Step One Plus™ Real-Time PCR System. Raw data analyses were conducted by corresponding setup software to get averaged Cycle Threshold (CT) values from the triplicates data. Relative gene expression quantification was done with Microsoft Excel 2013 following the 2- ΔΔCT method [16,17].

Table S3 RT-qPCR primers

Target gene	Stock Number	Sequence / Identity	Reference
GFP	RKO722	QT01079589	www.qiagen.com
RpL32	RKO677	QT00985677	www.qiagen.com
Act5c	RKO744<br>RKO745	5` GTGCACCGCAAGTGCTTCTAA 3`<br>5` TGCTGCACTCCAAACTTCCAC 3`	[14]
Stim-RNAi RNA	YXO1119<br>/YXO1120	5` GAGAACTCTGAATAGGGAATTGGG 3`<br>5' ACACTTCCACTGTAGCAATCAA 3'	This study
Stim	RWO713	QT00923020	www.qiagen.com
AkhR siRNA	YXO1194	5` TCCGCTGATCATGTTCATCTA 3`	www.thermofisher.com<br>4440418
AkhR	RKO719	QT00931210	www.qiagen.com
Akh	MGO1009	QT00957859	www.qiagen.com
bmm	YXO1169	QT00964460	www.qiagen.com
CG7461 (dACADVL)	RKO746<br>RKO747	5' CTGCCACATCCAGTCATCATAG 3'<br>5' GATCCAAGATTCCAGTCGTAGTC	This study

		3’	
CPT1	YXO1155<br>YXO1156	5’ TTCTCAACTTCCGTCGACTTATC 3’<br>5’ GTACGTTCATACTGCCAGGAG 3’	This study
blw (dATP5A)	YXO1161<br>YXO1162	5’ AGCGCTGAGATCTCCAACAT 3’<br>5’ TGTCGGGCTCCAAGTTAAG 3’	This study
ATP synβ (dATP5B)	YXO1163<br>YXO1164	5’ CGTCGATGTCCAGTTTGATG 3’<br>5’ AGTGCGCACGGTATTTTCTC 3’	This study
dFAS	YXO1187<br>YXO1188	5’ CTCCACCATCGAGGAGTTCA 3’<br>5’ CTTGAGCTTGCCAATCCTGT 3’	[18]
dACS	YXO1189<br>YXO1190	5’ TGTCGCATTTGAAGCGAGTGA 3’<br>5’ CCACCAGTAATCGCGGTCAT 3’	Fly Primer Bank: PP26971
Lipin	YXO1191<br>YXO1192	5’ CACACCGACAACACACTGGA 3’<br>5’ CTTCTTCTCGCCCTGAAACAG 3’	Fly Primer Bank: PP22555
sug	YXO1191<br>YXO1192	5’CCAGTCCGTGATCATGAAGGCTC3’<br>5’GTGCCAGTGCATCCAAGGTGTCG3’	[18]
aralar1	YXO1195<br>YXO1196	5’ TCCTGGGACTCTTTTCCGAATC 3’<br>5’ GCCTGGAACTCCGAGAAGGA 3’	This study
Mdh1	YXO1181<br>YXO1182	5’ GGAATGGAGCGAAAGGATCTG 3’<br>5’ GCACCTTGACGTCCTTCTTG 3’	This study
Mdh2	YXO1197<br>YXO1198	5’ CGCCGATCTGTCGCATATC 3’<br>5’ CACCGGGTTGGTGATGATGG 3’	This study
Got1	YXO1199<br>YXO1200	5’ TAACCACGAGTATTTGCCAGTG 3’<br>5’ ACATTGCGATTCAGTTGTGTGT 3’	This study
Got2	YXO1201<br>YXO1202	5’ TGTCACGGAAGCCTTCAAGAA 3’<br>5’ GTCCAGACTACGGCTCACCA 3’	This study
Hex-C	YXO1203<br>YXO1204	5’ GCGGAGGTGCGAGAACTTAT 3’<br>5’ CCACTTCCAGGCAAAAGCGA 3’	Fly Primer Bank: PP11731
CCHa2	YXO1075<br>YXO1076	5’ CCCGTCAGGTGCTTTACAAA 3’<br>5’ CGGAATTGGCCAAGGGATAA 3’	[19]
daw	YXO1185<br>YXO1171	5’ CAAGCGGGAGTACTATGCCC 3’<br>5’ CCGGGATGGTTGTAGCTGAG 3’	[18]
ImpL2	YXO1042<br>YXO1043	5’ AAGAGCCGTGGACCTGGTA 3’<br>5’ TTGGTGAACTTGAGCCAGTCG 3’	Based on [20]
Lst	RKO1106	QT00948185	www.qiagen.com

For Taqman RT-qPCR, 15 adult male fly abdomen of 4 independent biological replicates were collected for total RNA extraction with QIAGEN miRNA extraction kit (Qiagen, Cat. #217004). *AkhR* siRNA-specific RT primer set, *AkhR* siRNA specific qPCR primer set, and *AkhR* siRNA specific TaqMan probes (Thermo Fisher Scientific, Custom TaqMan® Small RNA Assay, Cat. #4440418) were designed and produced by Applied Biosystems (Thermo Fisher Scientific,

www.thermofisher.com) based on *AkhR* shRNA RNAi transgene targeted sequence: TCCGCTGATCATGTTCATCTA[21]. *AkhR* siRNA-specific RT primer set, 2s rRNA (Applied Biosystems, Thermo Fisher Scientific, Cat. #4427975)-specific primer set, TaqMan MicroRNA Reverse Transcription Kit (Applied Biosystems, Thermo Fisher Scientific, Cat. #4366596) were used for reverse transcription, and TaqMan Universal PCR Master Mix II (Applied Biosystems, Cat. #4440040) were used for qPCR. The protocol of TaqMan Small RNA Assays (Thermo Fisher Scientific, www.thermofisher.com) was followed for TaqMan RT-qPCR experiments.

Food intake assay

Food intake assays were performed in so called CAFE plates, i.e. modified 24-well cell culture plates. The bottom of each well was replaced with a metal net to allow air ventilation. A silicone layer was placed between the plate lid and the plate body and appropriate holes were inserted into the lid of the plate, to hold a single ring caps capillary per well (Hirschmann, Eberstadt, Germany, Cat. #: 9600105) loaded with liquid food (5% sucrose, 5% yeast enzymatic extract (MP Biomedicals, Santa Ana, USA, Cat. #103304) in H_2O). Each capillary was loaded with around 5 µL liquid food. After each capillary was inserted into the individual chamber, which housed a single male fly, the liquid food level was marked. On each plate, one empty well served as evaporation control to be subtracted from the food consumption of the flies. After one day, food level reduction in each fly well and the evaporation controls were recorded daily (ruler and/or camera imaging) to calculate the food intake volume based on the length of food level change marked on the capillary. The CAFE plates were kept at corresponding temperature in a sealed box with diurnal day/night cycle (12h/12h) in an incubator. High humidity was provided by a water layer at bottom of the sealed box. For *Stim*-TRiPI experiments two day old flies were transferred into CAFE chambers to adapt to the new environment for two days prior to recording. Then, their daily food intake was monitored before *Stim*-TRiPI, during *Stim*-TRiPI and after *Stim*-TRiPI treatment. For *Stim*-DRiPI flies, their daily food intake was monitored following *Stim*-DRiPI pulse induction. Data processing and statistical analysis were performed with Fiji (ImageJ, 1.51h) and Excel 2011 (Student's t-test). Experiments were repeated twice.

Starvation resistance assay

Day 21 *Stim*-TRiPI On/Off, *Stim*-TRiPI On control and Stim-TRiPI Off control male flies were used for starvation resistance experiments. In each experiment, at least 200 flies were kept in 10 small-size vials (each vial contains around 20 flies). Each vial was filled with 10 mL 1% agarose for water supply. The assay was done at 18°C with a diurnal-light/dark cycle. After 48h of starvation, 20 flies of each experimental fly batch were collected and frozen at -20°C for later CCA assay. Starved flies, which died during early starvation, were counted and removed three times a day (i.e. in the morning, in the afternoon, and the evening). The flies,

which died after the median survival time were collected and stored at -20°C for later CCA assay. Data processing and survival analysis were performed with Excel 2011 and online survival analysis software OASIS (Log Rank test) [22]. Experiments, which were repeated twice.

Startle-induced climbing assay

Startle-induced climbing assays were performed as described in Gáliková et al., 2015 [5]. At least 250 *Stim*-TRiPI On/Off male flies at day 21 after *Stim*-TRiPI were distributed into eight small-size vials (each vial contains around 30 flies) one day prior to testing. The climbing assay was performed using the modified 'countercurrent distribution' method [23,24]. Flies were repeatedly tapped onto the bottom of assay tubes. The climbing ability of adult male flies was assayed by counting the number of flies which climbed a defined height within a limited time period (30s-1min). The climbing index was calculated as described in Gáliková et al., 2015 [5]. Experiments were repeated at least twice.

Lifespan assay

At least 200 *Stim*-DRiPI On and *Stim*-DRiPI Off male flies (based on drug inducible GeneSWITCH system) were distributed at day 0 into 15-18 small-size vials (each vial contained 15 flies as one replicate) for lifespan assay. Each vial contained 5 mL Gö-food. The lifespan assay was carried out at 25°C with diurnal-light/dark cycle (12h/12h) and 60% relative humidity in an incubator. The fly vials were placed horizontally. The flies were transferred every two days into new vials with fresh food without CO_2 anaesthetization. Dead flies were counted and removed every other day before and daily after flies reached median survival time. Data processing and lifespan survival analysis were performed with Excel 2011 and online survival analysis software OASIS (Log Rank test). Experiments were repeated twice.

Metabolic rate assay

Fly metabolic rate was estimated based on a modification of the respirometry method described previously [25]. Cohorts of five adult flies were assayed over 2-3h at noon and in the evening. The CO_2 production rate was quantified in a self-made assay tube composed of a 1 mL pipette tip. The tip end of the pipette was attached to a capillary (BLAUBRAND®, 25µL, Brand, Wertheim, Germany, Cat. #: 708722), and tightly sealed with glue (Power-Pritt-Gel, Henkel, Düsseldorf, Germany). The pipet tip was then loaded with a small piece of sponge foam and loaded with soda lime (around (1/4 volume of the pipet tip) (Sigma-Aldrich, Cat. #72073-1KG). On the top of the soda lime, another small piece of sponge foam was placed to isolate the flies from the lime. Five male flies of a given experimental series were transferred into the assay tube, which was top-sealed with modelling clay to close the system air-tight. Fly-emitted CO_2 was absorbed by the soda lime to create low pressure. One assay tube housing

five dead flies was used as a metabolic rate negative control. To ensure a constant environment such as constant humidity (60%), constant temperature (25°C), the assay device was placed in an incubator. The assay tubes containing the flies were set into a frame in the sealed glass chamber. The bottom of glass chamber was filled with a diluted, red coloured Eosin-solution (Sigma-Aldrich, Cat. #:HT110232-1I) into which the capillary end of the assay tube was immersed. The increasing volume of the Eosin solution in the capillaries was monitored every 30min by camera imaging (FujiFilm X-T10, FujiFilm, Tokyo, Japan). Assay were done both at noon and in the evening (technical replicates). Images were processed with Fiji (ImageJ, 1.51h). Statistical analysis was performed with Excel 2011. Experiments were repeated twice.

RNAseq, gene differential expression and gene ontology analysis

RNA was prepared as described above and RNA quality was controlled using an Agilent 2100 Bioanalyzer (Agilent Technologies, Santa Clara, United States). PolyA-containing mRNAs of each sample were enriched, fragmented, and then reverse transcribed into adaptor-barcoded cDNA libraries using the TruSeq Stranded mRNA Library Prep Kit (Illumina). Barcoded libraries from three biological replicates per condition were normalized and pooled, which were subsequently sequenced with Illumina HighSeq2500 (100 bp paired end reads, and around 30 million sequencing depth) to generate (raw sequencing file data: GSE112689). Sequencing data were filtered, trimmed and mapped to the annotated *Drosophila melanogaster* genome (dmel-all-gene-r6.06.fasta.gz, FlyBase, http://flybase.org/), which allows counting reads per genes by CLC bio (Qiagen, Hilden, Germany) (raw reads count file, supplementary data file 1). RNAseq dataset from Akh overexpressed fly larval fat body and control fly fat body is GSE92350 [26]. Raw reads count data of two RNAseq dataset were processed by DESeq2 [27] for gene differential analysis.

DESeq2 analysis was done based on the vignette as well as one workflow (http://www.huber.embl.de/users/klaus/Teaching/DESeq2Predoc2014.html). After log transformation of raw reads of each samples with DESeq2, clustering analysis (based on Euclidean distances) and principal component analysis (PCA) were further performed and visualized with R (R Core Team, 2016) and R studio (RStudio, 2015) [28]. Genes expressed with a log2fold change >0.5 and <-0.5, adjusted p value<0.1 were classified as differentially expressed genes (Wald test, p value adjusted with Benjamini-Hochberg method) (differential expressed gene file, supplementary data file 2). Differential expressed genes identified above (up-regulated and down-regulated) were further analysed by DAVID: Functional Annotation Clustering (https://david.ncifcrf.gov). genes classified as “Secreted” were listed in subsheet of supplementary data file 2. Moreover, literature study also identify *CCHa2* [29,30], *Lst* [31] as genes encoding candidate secreted hormones.

Gene Ontology (GO) (biological process) enrichment analyses of down-regulated genes were carried out by FlyMine [32] with adjusted p value < 0.05 (http://www.flymine.org/). Enriched GO terms with the corresponding p values were summarized and clustered based on semantic similarities by REVIGO with default setting [33] to generate R scripts for visualization. R session information refer to supplementary data file 3. Corresponding specified R scripts mentioned above is shared on Github: https://github.com/kfJan/-Gene-differential-expression-analysis-by-DESeq2-and-visualisation-of-adult-fly-fat-body-RNAseq-data. Biochemical pathways are illustrated by PathVisio [34,35]. Intersection analysis, Venn diagram and heatmap of commonly regulated genes by *Akh* overexpression and *Stim*-TRiPI On were done by R, R package-VennDiagram (1.6.20 [36]) and pheatmap (1.0.20 [37]).

Western blot analysis

Fifteen fly abdomen per replicate (three replicates) were homogenized in 300 µL tissue lysis buffer and protein concentrations were determined using the Bradford assay (Sigma-Aldrich, Cat. #B6916_500). Tissue lysis buffer composition for Western blot analysis was kindly provided by Dr. Yuanbin Xie (Tris-HCl 250mM, NaCl 125mM, glycerol 5% (v/v), IGEPAL CA-630 (Sigma-Aldrich, Cat. #I8896-50ML) 0.5% (v/v), $MgCl_2$ 1.5 mM, Na-deoxycholate (0.25%, v/v), Dithiothreitol (DTT) 1mM, NaF 25mM, Na_3VSO_4 1mM, ethylene glycol-bis(β-aminoethyl ether)-N,N,N',N'-tetraacetic acid (EGTA) 2mM, EDTA 1mM, Phosphatase inhibitors 1x (Roche, Basel, Switzerland, Cat. #04906837001)).

Protein samples were adjusted to a concentration of 7 µg/µL by adding lysis buffer. The normalized protein samples were mixed with an equal volume of 2x sample buffer (final 1x; Stock 3x: 150mM Tris [pH 6.8], 6% Sodium dodecyl sulphate (SDS), 0.3% Bromophenol blue, 30% Glycerol, 300mM DTT and incubated at 95°C for 3min followed by centrifugation (13,000 rpm for 3min). Around 15 µL of the supernatant (contains about 50 µg protein) and 10 µL of a PageRuler prestained protein ladder (Thermo Fisher Scientific, Cat. #26616) were loaded, separated, transferred (BioRad system: 10% SDS-PAGE gel, 70V 30min + 150V 60min and wet-electro transfer: 300mA, 1 h on ice) to nitrocellulose membrane by standard Western blot procedure. Blots were incubated with the following diluted primary antibodies (1xTBST+5%BSA) at 4°C overnight: rabbit anti-Phospho-Akt Ser505 (1:500, NEB, Cat. #405S), rabbit anti-Phospho-Akt Thr342 (1:500, PhosphoSolutions, Cat. #:104-342), rabbit anti-Akt (1:500, NEB, Cat. #:9272S) and mouse anti-tubulin (Developmental Studies Hybridoma Bank (DSHB), Cat. #E7) in a successive order with washing, striping (ThermoFisher Scientific, Cat. #:46430), and blocking step in between; secondary antibody solutions (1xTBST+5%BSA) at room temperature for 1h: goat anti-rabbit-HRP (1:2000, Pierce, Cat. #31460) or goat anti-mouse-HRP (1:10000, Pierce Cat. #31430). The membrane was finally incubated for 3min in Western™ Bright quantum chemiluminescent reagents- mixed

solution (Biozym, Cat. #K-12042-D10) at room temperature in the dark. The chemiluminescence of horseradish peroxidase (HRP)-conjugated secondary antibody/primary antibody/antigen protein complexes was detected by either a Fujifilm LAS-1000 CH Plus CCD Camera System. Images were processed and analysed by Fiji (ImageJ, 1.51h) or film developing system.

Table S4 Sample information in Western blot whole gel image represented in S11.

number	Genotype information
1	*Stim*-DRiPI Off adult male abdomen biological replicate 35
2	*Stim*-DRiPI On adult male abdomen biological replicate 35
3	*Stim*-DRiPI Off adult male abdomen biological replicate 37
4	*Stim*-DRiPI On adult male abdomen biological replicate 37
5	*Stim*-DRiPI Off adult male abdomen biological replicate 39
6	*Stim*-DRiPI On adult male abdomen biological replicate 39
7	*Stim*-DRiPI Off adult male abdomen biological replicate 44
8	*Stim*-DRiPI On adult male abdomen biological replicate 44
9	*Stim*-DRiPI Off adult male abdomen biological replicate 46
10	*Stim*-DRiPI On adult male abdomen biological replicate 46
11	*Stim*-DRiPI Off adult male abdomen-replicate 86 under 12hs starvation + 2hs refeeding
12	*Stim*-DRiPI Off adult male abdomen replicate 86 after 12hs starvation

Akh and dIlp2 immunostaining and confocal imaging

Prior to the immunostaining, the brain, the corpora cardiaca, and the ventral cord of adult males were dissected in cold 1xPBS (phosphate-buffered saline) and fixed in 3.7% paraformaldehyde on ice for 2 h (eight to twelve replicates). Then, tissues were washed with 0.25% TritonX-100 (Merck, Darmstadt, Germany) in PBS (PBST) for 30min at room temperature and blocked with 1% Bovine serum albumin (BSA) and 3% Normal Goat Serum (NGS) (Invitrogen, Thermo Fisher Scientific, Cat. #31873) in 0.25% PBST at room temperature for 1h. Afterwards, tissues were incubated with the respective primary antibodies at 4°C overnight: rabbit anti-Akh (diluted 1:300 [38]), rat anti-dIlp2 (diluted 1:400 [39]) and mouse anti-nc82 (diluted 1:50) (DSHB, Cat. #nc82). After 3x10 minutes washing with 1%BSA and 3%NGS in 0.25% PBST at room temperature, tissues were incubated with secondary antibodies: Alexa-568-goat anti-rabbit (diluted 1:1000, Invitrogen, Thermo Fisher Scientific, Cat. #A-11036), Alexa-488-goat anti rat (diluted 1:1000, Invitrogen, Thermo Fisher Scientific, Cat. #A-11006), and anti-Alexa-647-goat anti-mouse (diluted 1:1000, Jackson ImmunoResearch Laboratories, Cat. # 115-607-186). The stained tissues were washed 5x10 minutes in PBST at room temperature and kept

overnight in PBS at 4°C for extra washing. Fixed tissues were mounted in Mowiol (Sigma-Aldrich, Cat. #32,549_0). For confocal imaging, the same confocal system as described above with a Zeiss Plan Apochromat 40x/1.4 oil DIC M27 objective was used. The averaged Akh staining fluorescence intensities were quantified using Z-axis maximum intensity projections of image stacks. Image analysis was performed with Fiji (ImageJ, 1.51h). Statistical analysis was done with Excel 2011 (Student's t-test). Experiments were repeated twice.

Circulating sugar level measurement

The hemolymph collection of adult fly and circulating sugar measurement is based on the method described previously [5]. 30 flies (one sample replicate, 8 replicates in total for each genotype) were decapitated and fly bodies were transferred to one 0.5 ml tube with a piece of cotton wool and 4 holes in the bottom (created with a needle). The 0.5ml tube was further put into a 1.5ml Eppendorf tube and centrifuged with 9000 rcf at 4°C for 6 min. 1 µl collected hemolymph in the 1.5 ml tube was mixed with 99µl 0,05% Tween-20, and incubated at 70°C for 5min for circulating sugar measurement: 30µl per sample of the diluted hemolymph was reacted with 100 µl of the GO reagent + 0.3 µl of porcine trehalase (Sigma, T8778-1U) at 37°C for overnight. Reaction was stopped by adding 100 µl of 6N H_2SO_4. Total circulating sugar (Glucose + Trehalose) was determined based on the relative absorption (OD at 540nm) of samples and glucose standards (0, 0.02, 0.04, 0.06, 0.08, 0.1 µg /µl).

EdU staining

Flies were fed with Gö-food containing EdU (100µM) from day 5 to day 11 after *Stim*-TRiPI On or Off. Incorporation of EdU during DNA synthesis was detected through the EdU reaction with an azide fluorescent dye (Alexa). EdU staining was carried out with the Click-iT® EdU Alexa Fluor® 647 Imaging Kit according to manufacture instruction (Molecular probes, ThermoFisher Scientific, Cat. #: C10340). Briefly, dissected fly tissues were fixed with 3.7% paraformaldehyde in PBS solution at room temperature for 20 min. After washing with 3% BSA (Biomol, Cat. #: 014001) in PBS, the fixed tissues were permeabilized with 0.5% Triton® X-100 (Merck) in PBS (PBST) for 20 min at room temperature. Afterwards, having washed with 3% BSA in PBS, fly tissues were incubated with Click-IT reaction cocktail at room temperature for 30 min in the dark. After washing, cell nuclei in the tissue were stained with Hoechst 33342 (5 µg/mL) in PBS. The tissues were washed and embedded in Mowiol medium for confocal imaging. For confocal imaging, and processing of the images see above Akh and dIlp2 staining part. The excitation/emission peak wavelength of EdU and Hoechst staining dyes are 647/665 nm and 350/461 nm, respectively.

Circulating dIlp2HF level determination

Measuring circulating dIlp2HF levels is based on the method reported in Park et al [40] and Li et al [41]. The Stim-DRiPI flies were crossed with flies containing FLAG- and HA- tagged version of dIlp2 (*Ilp2*^{1} , *gd2HF(attP2))* in 3rd chromosome. FLAG(GS)HA peptides standard dissolved in PBS (0.156, 0.312, 0.625, 1.25, 2.5, 5, 10, 20ng/μl) were used for standard curve. Bottom end of day 4 *Stim*-DRiPI Off and On adult male flies' abdomens were cut with a small scissor. Haemolymph isolation and ELISA were carried out by following the protocol provided by Dr. Sangbin Park.

Supplementary information references

1. McGuire, S. E. Spatiotemporal rescue of memory dysfunction in *Drosophila*. *Science*. **302,** 1765–1768 (2003).

2. Tricoire, H. *et al*. The steroid hormone receptor EcR finely modulates *Drosophila* lifespan during adulthood in a sex-specific manner. *Mech. Ageing Dev*. **130,** 547–552 (2009).

3. Beller, M. *et al*. PERILIPIN-dependent control of lipid droplet structure and fat storage in *Drosophila*. *Cell Metab*. **12,** 521–532 (2010).

4. Eid, J.-P., *et al*. The *Drosophila* STIM1 orthologue, dSTIM, has roles in cell fate specification and tissue patterning. *BMC Dev. Biol*. **8,** 104 (2008).

5. Gáliková, M. *et al*. Energy homeostasis control in *Drosophila* adipokinetic hormone mutants. *Genetics* **201,** 665–683 (2015).

6. Grönke, S., *et al*. Molecular evolution and functional characterization of *Drosophila* insulin-like peptides. *PLoS Genet*. **6,** e1000857 (2010).

7. Rubin, G. M. *et al*. A *Drosophila* complementary DNA resource. *Science*. **287,** 2222–2224 (2000).

8. Bischof, J., *et al*. An optimized transgenesis system for *Drosophila* using germ-line-specific phiC31 integrases. *Proc. Natl. Acad. Sci. U. S. A.* **104,** 3312–3317 (2007).

9. Spandl, J., *et al*. Live cell multicolor imaging of lipid droplets with a new dye, LD540. *Traffic* **10,** 1579–1584 (2009).

10. Schindelin, J. *et al*. Fiji - an Open Source platform for biological image analysis. *Nat. Methods* **9,** 676–82 (2012).

11. Schindelin, J., *et al*. The ImageJ ecosystem: an open platform for biomedical image analysis. *Mol Reprod Dev*. **82,** 518–529 (2015).

12. Baumbach, J., *et al*. Gαq, Gγ1 and Plc21C control *Drosophila* body fat storage. *J. Genet. Genomics* **41,** 283–292 (2014).

13. Kaluzny, M. A., *et al*. Rapid separation of lipid classes in high yield and purity using bonded phase columns. *J. Lipid Res*. **26,** 135–140 (1985).

14. Bauer, R. *et al*. Schlank, a member of the ceramide synthase family controls growth and body fat in *Drosophila*. *EMBO J*. **28,** 3706–3716 (2009).

15. Ponton, F., *et al*. Evaluation of potential reference genes for reverse transcription-qPCR studies of physiological responses in *Drosophila melanogaster*. *J. Insect Physiol*. **57,** 840–850 (2011).

16. Livak, K. J. & Schmittgen, T. D. Analysis of relative gene expression data using real-time quantitative PCR and the 2(-Delta Delta C(T)) Method. *Methods* **25,** 402–408 (2001).

17. Schmittgen, T. D. & Livak, K. J. Analyzing real-time PCR data by the comparative CT method. *Nat. Protoc*. **3,** 1101–1108 (2008).

18. Mattila, J. *et al*. Mondo-Mlx mediates organismal sugar sensing through the Gli-similar transcription factor sugarbabe. *Cell Rep*. **13,** 350–364 (2015).

19. Gáliková, M., *et al*. The obesity-related adipokinetic hormone controls feeding and expression of neuropeptide regulators of *Drosophila* metabolism. *Eur. J. Lipid Sci. Technol*. **119,** 1600138 (2017).

20. Kwon, Y. *et al*. Systemic organ wasting induced by localized expression of the secreted insulin / IGF antagonist ImpL2. *Dev. Cell* **33,** 36–46 (2015).

21. Perkins, L. A. *et al*. The transgenic RNAi project at Harvard medical school: resources and validation. *Genetics* **201,** 843–852 (2015).

22. Yang, J. S. *et al*. OASIS: online application for the survival analysis of lifespan assays performed in aging research. *PLoS One* **6,** e23525 (2011).

23. Benzer, S. Behavioral mutants of *Drosophila* isolated by countercurrent distribution. *Proc. Natl. Acad. Sci. U. S. A*. **58,** 1112–1119 (1967).

24. Shcherbata, H. R. *et al*. Dissecting muscle and neuronal disorders in a *Drosophila* model of muscular dystrophy. *EMBO J*. **26,** 481–493 (2007).

25. Yatsenko, A. S., *et al*. Measurement of metabolic rate in *Drosophila* using respirometry. *J. Vis. Exp*. **88,** e51681 (2014).

26. Song, W. *et al*. Midgut-derived Activin regulates glucagon-like action in the fat body and glycemic Control. *Cell Metab*. **25,** 386–399 (2017).

27. Love, M. I., Huber, W. & Anders, S. Moderated estimation of fold change and dispersion for RNA-seq data with DESeq2. *Genome Biol*. **15,** 550 (2014).

28. RStudio Team. RStudio: Integrated Development for R. (2015).

29. Ren, G. R. *et al*. CCHamide-2 Is an orexigenic brain-gut peptide in *Drosophila*. *PLoS One* **10,** e0133017 (2015).

30. Sano, H. *et al*. The nutrient-responsive hormone CCHamide-2 Controls growth by regulating insulin-like peptides in the brain of *Drosophila melanogaster*. *PLoS Genet*. **11,** 1–26 (2015).

31. Alfa, R. W. *et al*. Suppression of insulin production and secretion by a decretin hormone. *Cell Metab*. **21,** 323–333 (2015).

32. Lyne, R. *et al*. FlyMine: an integrated database for *Drosophila* and *Anopheles* genomics. *Genome Biol*. **8,** R129 (2007).

33. Supek, F., *et al*. Revigo summarizes and visualizes long lists of gene ontology terms. *PLoS One* **6,** e21800 (2011).

34. van Iersel, M. P. *et al*. Presenting and exploring biological pathways with PathVisio. *BMC Bioinformatics* **9,** 1–9 (2008).

35. Kutmon, M. *et al*. PathVisio 3: An Extendable Pathway Analysis Toolbox. *PLoS Comput. Biol.* **11,** 1–13 (2015).

36. Chen, H. & Boutros, P. C. VennDiagram : a package for the generation of highly-customizable Venn and Euler diagrams in R. *BMC Bioinformatics* **12,** http://www.biomedcentral.com/1471-2105/12/35 SOFTW (2011).

37. Kolde, R. Pheatmap: pretty heatmaps-R package. (2012).

38. Kim, S. K. & Rulifson, E. J. Conserved mechanisms of glucose sensing and regulation by *Drosophila* corpora cardiaca cells. *Nature* **431,** 316–320 (2004).

39. Géminard, C., Rulifson, E. J. & Léopold, P. Remote control of insulin secretion by fat cells in *Drosophila*. *Cell Metab.* **10,** 199–207 (2009).

40. Park, S. *et al*. A genetic strategy to measure circulating *Drosophila* insulin reveals genes regulating insulin production and secretion. *PLoS Genet.* **10,** e1004555 (2014).

41. Li, Y. *et al*. Octopamine controls starvation resistance, life span and metabolic traits in *Drosophila*. *Sci. Rep.* **6,** 35359 (2016).
